# Polyfunctional CD4 T-cells correlating with neutralising antibody is a hallmark of COVISHIELD^TM^ and COVAXIN^®^ induced immunity in COVID-19 exposed Indians

**DOI:** 10.1038/s41541-023-00731-w

**Published:** 2023-09-14

**Authors:** Srabanti Rakshit, Sudhir Babji, Chaitra Parthiban, Ramya Madhavan, Vasista Adiga, Sharon Eveline J, Nirutha Chetan Kumar, Asma Ahmed, Sudarshan Shivalingaiah, Nandini Shashikumar, Mamatha V, Avita Rose Johnson, Naveen Ramesh, Ramkrishna Goud B, Mangaiarkarasi Asokan, Satyajit Mayor, Gagandeep Kang, George D’souza, Mary Dias, Annapurna Vyakarnam

**Affiliations:** 1 Division of Infectious Diseases, St. John’s Research Institute, Bangalore, Karnataka India; 2 The Wellcome Trust Research Laboratory, Christian Medical College, Vellore, Tamil Nadu India; 3 Department of Biotechnology, PES University, Bangalore, Karnataka India; 4 St. John’s Medical College, Bangalore, Karnataka India; 5 National Centre for Biological Sciences, Bengaluru, Karnataka India; 6 Department of Pulmonary Medicine, St. John’s Medical College, Bangalore, Karnataka India; 7 Department of Immunobiology, School of Immunology & Microbial Sciences, Faculty of Life Science & Medicine, King’s College, London, UK

**Keywords:** Inactivated vaccines, Viral infection

## Abstract

Detailed characterisation of immune responses induced by COVID-19 vaccines rolled out in India: COVISHIELD^TM^ (CS) and COVAXIN® (CO) in a pre-exposed population is only recently being discovered. We addressed this issue in subjects who received their primary series of vaccination between November 2021 and January 2022. Both vaccines are capable of strongly boosting Wuhan Spike-specific neutralising antibody, polyfunctional Th1 cytokine producing CD4+ T-cells and single IFN-γ + CD8+ T-cells. Consistent with inherent differences in vaccine platform, the vector-based CS vaccine-induced immunity was of greater magnitude, breadth, targeting Delta and Omicron variants compared to the whole-virion inactivated vaccine CO, with CS vaccinees showing persistent CD8+ T-cells responses until 3 months post primary vaccination. This study provides detailed evidence on the magnitude and quality of CS and CO vaccine induced responses in subjects with pre-existing SARS-CoV-2 immunity in India, thereby mitigating vaccine hesitancy arguments in such a population, which remains a global health challenge.

## Introduction

The COVID-19 pandemic declared by the WHO on 11^th^ March 2020, caused by the novel SARS-CoV-2 coronavirus has contributed to loss of millions of lives worldwide^1^. However, COVID-19 death toll was mitigated significantly by the early global roll out of several SARS-CoV-2 vaccines starting as early as December 2020^2,3^, many of which demonstrated remarkable effectiveness in reducing disease severity^4,5^. Vaccination in India was rolled out on 16^th^ January 2021 with the emergency use of two vaccines administered as a two-dose regimen^6^. The first one being introduced was the Oxford Astrazeneca/AZD1222 vaccine, a replication-deficient simian adenovirus–vectored (ChAdOx1 nCoV-19) vaccine, referred to as COVISHIELD^TM^, manufactured by Serum Institute of India with the second dose administered 3 months post the initial dose^7^. The second vaccine, COVAXIN® (BBV152), a whole-virion beta-propiolactone inactivated SARS-CoV-2 vaccine adjuvanted with a TLR7/8 agonist Imidazoquinolin gallamide (IMDG) adsorbed to alum was indigenously developed by Bharat Biotech Ltd, was rolled out in the second half of 2021, where the two doses were administered at a gap of 28 days^8^. Safety and immunogenicity studies demonstrate both vaccines to be efficacious^9–14^. In pooled data from four trials, AZD1222 had a protective efficacy of 67% for preventing symptomatic COVID-19 and nearly 100% for preventing hospitalization and severe infection^9,10^. In India, 98% of participants demonstrated seroconversion after the second dose of COVISHIELD^TM^ (CS), while vaccine effectiveness of one versus two doses in providing protection against COVID-19 infection, was 49% and 54% respectively^11,12^. In Phase I/II clinical trial, the seroconversion rates in COVAXIN® (CO) vaccinated individuals stood at 98.3% with 77.8% protection of vaccinated subjects against symptomatic infection; 93.4% from severe disease, and 63.6% from asymptomatic disease in corresponding Phase III trial^13,14^.

Previous studies have probed the magnitude of antibody and T-cell responses induced by these vaccines without ascertaining baseline seropositivity^15–17^. Hence, a systematic analysis of the qualitative aspects of cellular immune responses contextualised with the antibody response induced by two doses of both these vaccines as per the vaccination protocol in a population with a heterogenous pattern of pre-existing SARS-CoV-2 immunity with evidence of prior/ ongoing infection reflected by presence of circulating SARS-CoV-2 nucleocapsid binding antibodies in India, is lacking. In particular, few studies have probed the quality and breadth of T-cell immune responses, which are considered to play an important role in vaccine efficacy both in terms of T-cell help for antibody induction as well as an effector role in viral clearance^18–22^. Our study was exceptionally well poised to address this knowledge gap as an extension to a recent multi-centre study that we participated in, which provided longitudinal measurement of antibody responses to CS and CO vaccination in subjects deemed either seronegative or seropositive exclusively by the TrimericS IgG assay (an indirect chemiluminescence immunoassay used for the quantitative determination of neutralizing antibodies against the SARS-CoV-2 trimeric spike glycoprotein) and cellular analysis in subjects recruited right after the Delta wave^23^.

Our study provides a detailed immune analysis on the magnitude and quality of CS- and CO-induced T-cell responses using 17-colour multiparameter flow cytometry contextualised with the Ab response determined by the MSD platform in this unique population at two weeks post vaccination relative to matched baseline. Unequivocal data is provided that seroconversion induced by natural COVID-19 exposure does not negate significant expansion of polyfunctional SARS-CoV-2-specific CD4 + T-cells correlating with increased neutralising antibody titres following vaccination with two doses of either COVISHIELD^TM^ or COVAXIN®.

## Results

### Screening for baseline serostatus of subjects recruited to the study on the MSD platform confirmed majority to be SARS-CoV-2 seropositive

**Fig. 1:**
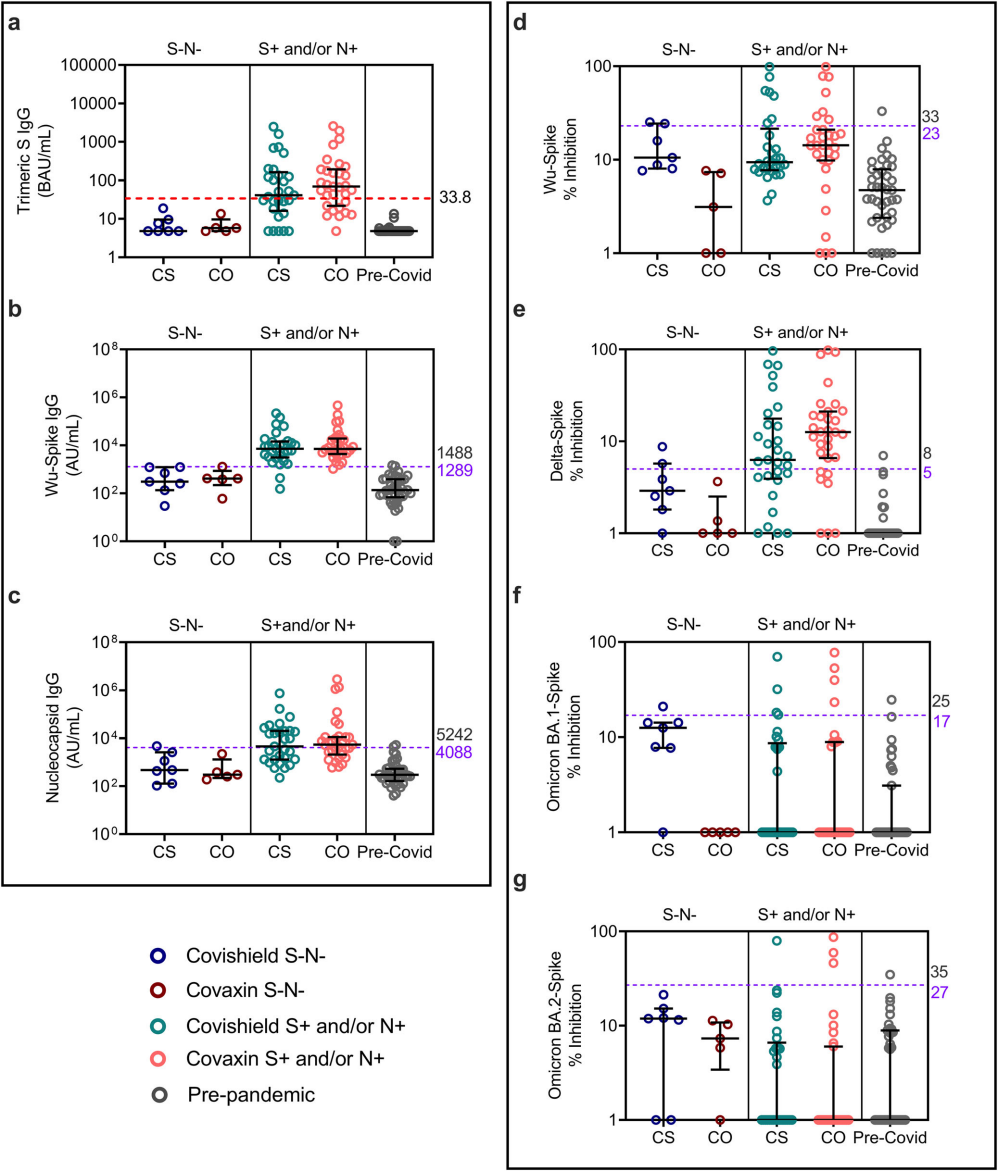
Antibody levels against SARS-CoV-2 at baseline after reclassification of COVISHIELD™ and COVAXIN® vaccinated individuals based on pre-pandemic antibody binding and pseudo neutralization surrogate assay cut-offs from Supplementary Fig. 3. **a** IgG against ancestral spike measured on the LIAISON® SARS-CoV-2 TrimericS IgG assay platform with an assay cut-off of 33.8 BAU/ml (horizontal red dotted line). **b**, **c** SARS-CoV-2 Spike and nucleocapsid IgG titres (AU/ml) measured by MSD V-PLEX COVID-19 Coronavirus Panel 1 (IgG). **d**–**g** Neutralizing antibody (% inhibition) to Wuhan, Delta and Omicron BA.1 and BA.2 variants measured by MSD V-PLEX SARS-CoV-2 Panel 25 (ACE2). Participants were classified as either S-N- [*n* = 7 for COVISHIELD^TM^ (dark blue); *n* = 5 for COVAXIN® (dark red)] or S+ and/or N+ [*n* = 29 for COVISHIELD^TM^ (teal), *n* = 31 for COVAXIN® (pink)] at baseline. Data are shown as median ± IQR.

A total of 120 subjects were recruited to the study at St John’s Research Institute, Bangalore. Of these 77 consented to receiving CS and 43 consented to receiving CO (Supplementary Fig. 1). Initial definition of baseline serostatus for downstream immunogenicity studies was based on the SARS-CoV-2 TrimericS IgG assay using a cut-off of 33.8 binding units per ml (BAU/ml) as per manufacturer’s recommendation. For a more comprehensive analysis of serostatus, pre-pandemic plasma collected in 2019 serving as negative control (Supplementary Fig. 2) and baseline samples from this study (Supplementary Fig. 3), were tested in parallel on the MSD platform for: Wuhan Spike (Wu-S) binding, Nucleocapsid (N) binding and for surrogate neutralising antibody (nAb) represented as percentage inhibition of ACE2-binding for variants of concern (VOC), including, ancestral (Wuhan), Delta, Omicron BA.1 and BA.2 strains. A cut-off for seropositivity was taken as being 3 SD above the pre-pandemic mean (Supplementary Fig. 2). Based on this analysis, it was noted that several seronegative samples on the TrimericS platform were seropositive on the MSD platform, with 58.8% of TrimericS negative samples showing binding antibody for Wuhan and 32.35% for N (Supplementary Fig. 3a–c). Of subjects with S binding antibody, 22% had nAb to Wuhan versus 61% to Delta, consistent with more recent exposure to the India Delta wave (between March and May 2021) compared to the ancestral Wuhan wave (between March and September 2020) (Supplementary Fig. 3d, e). Very few subjects had basal nAb to BA.1 or BA.2 (Supplementary Fig. 3f, g). Guided by this data, we redefined baseline serostatus (Supplementary Table 1) and replotted binding and neutralization antibody titre (Fig. 1). A minority of participants (22% and 16% recruited to receive CS and CO respectively) were seronegative and denoted as S and N double-negative (S-N-) (Fig. 1). The seropositive group had circulating binding antibodies to either S, N or both (Fig. 1a–c), the majority had basal nAb to Delta (Fig. 1d–g), highlighting the heterogenous nature of this group and were denoted as S+ and/or N+ henceforth.

### COVISHIELD^TM^ and COVAXIN® significantly enhance Wu-specific binding and neutralizing antibodies as well as CD4 + T-cell responses at 2 weeks post vaccination relative to pre-vaccine levels

**Fig. 2:**
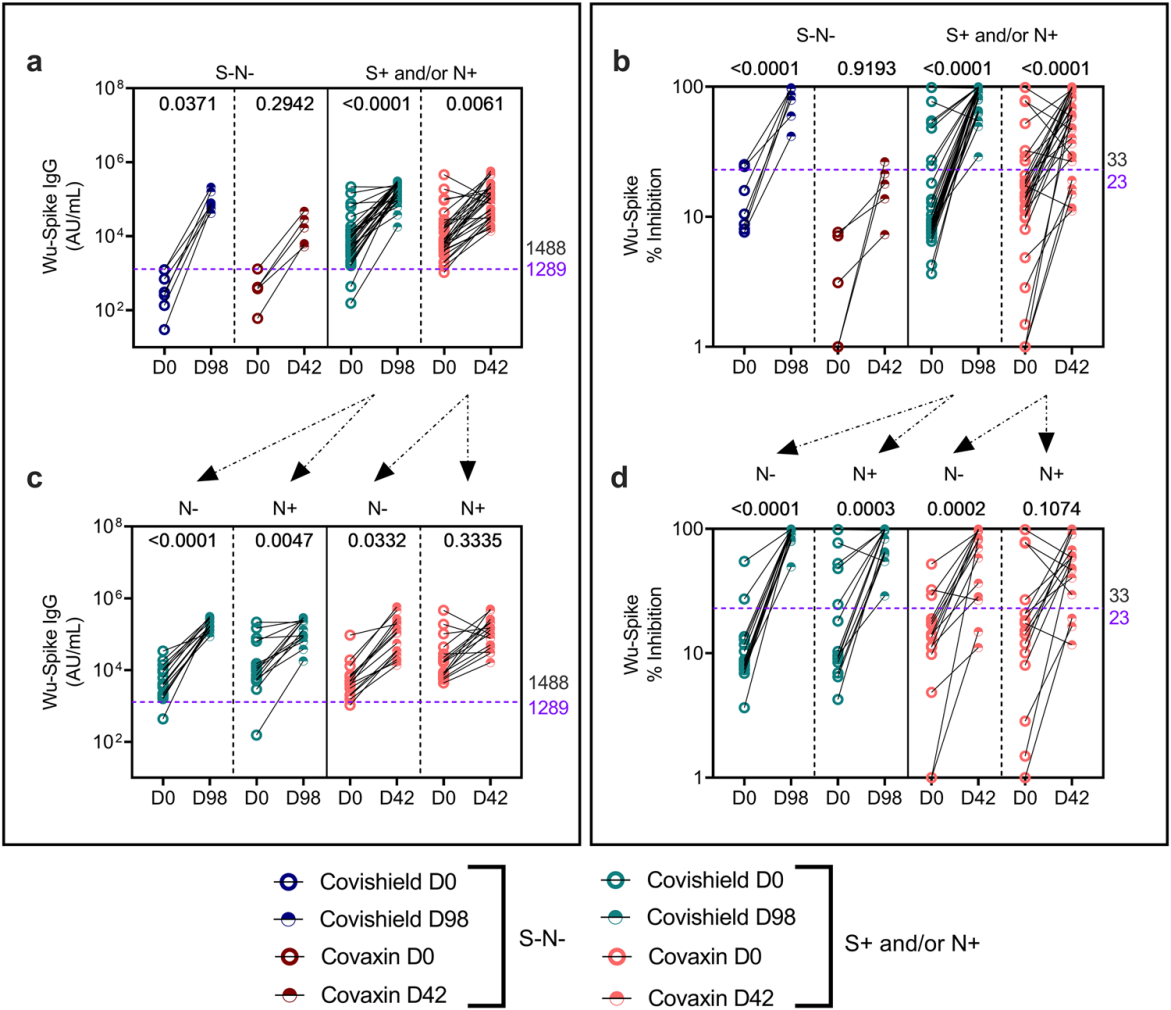
Vaccine-induced antibody responses to ancestral spike. **a** IgG levels (AU/ml) and **b** neutralizing antibody (% inhibition) against ancestral spike protein were measured in serum samples from S-N- [*n* = 7 for COVISHIELD^TM^ (dark blue); *n* = 5 for COVAXIN® (dark red)] or S+ and/or N+ [*n* = 29 for COVISHIELD^TM^ (teal), *n* = 31 for COVAXIN® (pink)] vaccinees by MSD V-PLEX SARS-CoV-2 panel 1 (IgG) and 25 (ACE2) respectively. **c** IgG levels (AU/ml) and **d** neutralizing antibody (% inhibition) against ancestral spike protein in subjects from the S+ and/or N+ group further segregated as N- (*n* = 15 for both COVISHIELD^TM^ and COVAXIN®) and N+ (n = 14 for COVISHIELD^TM^ and *n* = 16 for COVAXIN®). Line graphs represent antibody levels at baseline and 2 weeks post-vaccination. Assay cut-off values for each assay are denoted in purple and highest pre-pandemic cut-off in black. Two-way ANOVA with Šídák’s multiple comparisons test was used for statistical analysis.

**Fig. 3:**
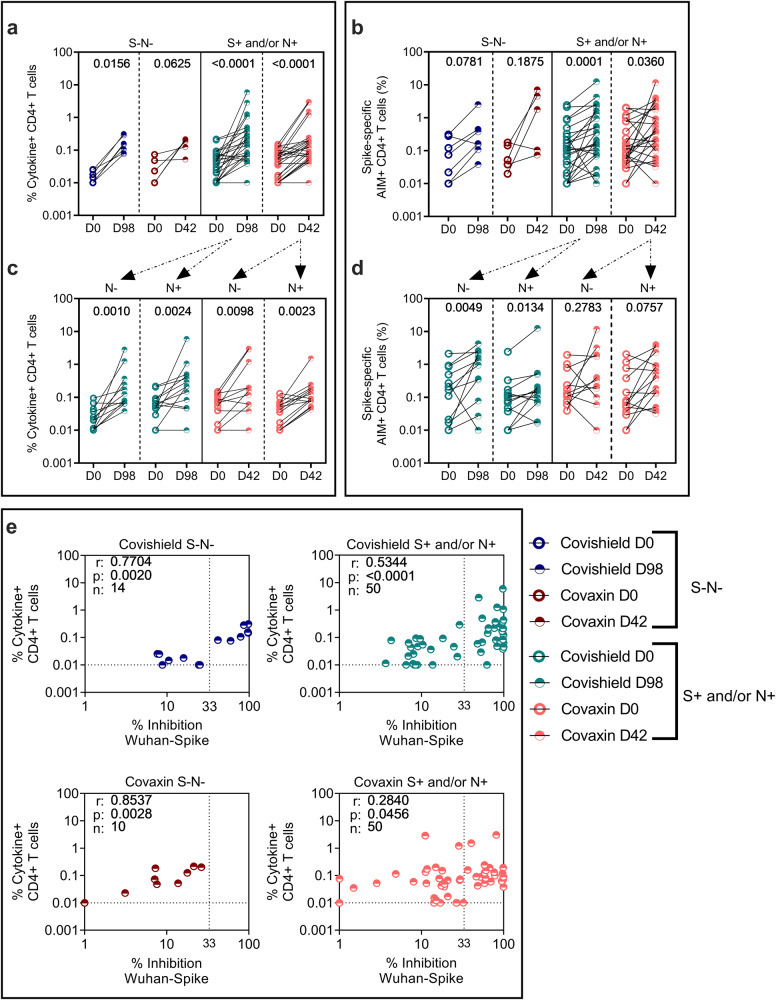
Vaccine-induced CD4 + T-cell responses to ancestral spike and comparative analysis of antibody and CD4 + T-cell responses in COVISHIELD^TM^ and COVAXIN® vaccinees. Whole Blood from COVISHIELD^TM^ and COVAXIN® individuals at baseline (D0) and 2 weeks post-vaccination (D98 for COVISHIELD^TM^ and D42 for COVAXIN®) were stimulated with spike peptide pool (1 µg/ml) for 20 h. CD4 + T-cells were analyzed for **a** intracellular expression of total cytokine+ cells (IFN-γ or IL-2 or TNF-a) obtained from Boolean gating in FlowJo and **b** for the frequencies of AIM expressing subsets (OX40 + CD137 + ). Participants were classified as either S-N- [*n* = 7 for COVISHIELD^TM^ (dark blue); *n* = 5 for COVAXIN® (dark red)] or S+ and/or N+ [*n* = 25 for COVISHIELD^TM^ (teal), *n* = 25 for COVAXIN® (pink)] at baseline. CD4 + T-cells expressing **c** effector cytokines and **d** AIM markers in subjects from the S+ and/or N+ group are further segregated as N- (*n* = 12 for COVISHIELD^TM^ and *n* = 11 for COVAXIN®) and N+ (*n* = 13 for COVISHIELD^TM^ and *n* = 14 for COVAXIN®). Line graphs represent background subtracted CD4 + T-cell frequencies. Statistical analyses was performed using a paired Wilcoxon test. **e** Correlations between total cytokine+ CD4 + T-cells (IFN-γ or IL-2 or TNF-a) in response to stimulation of whole blood with complete Wuhan spike peptide pool and corresponding neutralizing antibody responses (% inhibition) against SARS-CoV-2 Wuhan Spike at baseline and two weeks post-vaccination. Spearman’s correlation coefficient (r) and significance values (*P*) are indicated.

**Fig. 4:**
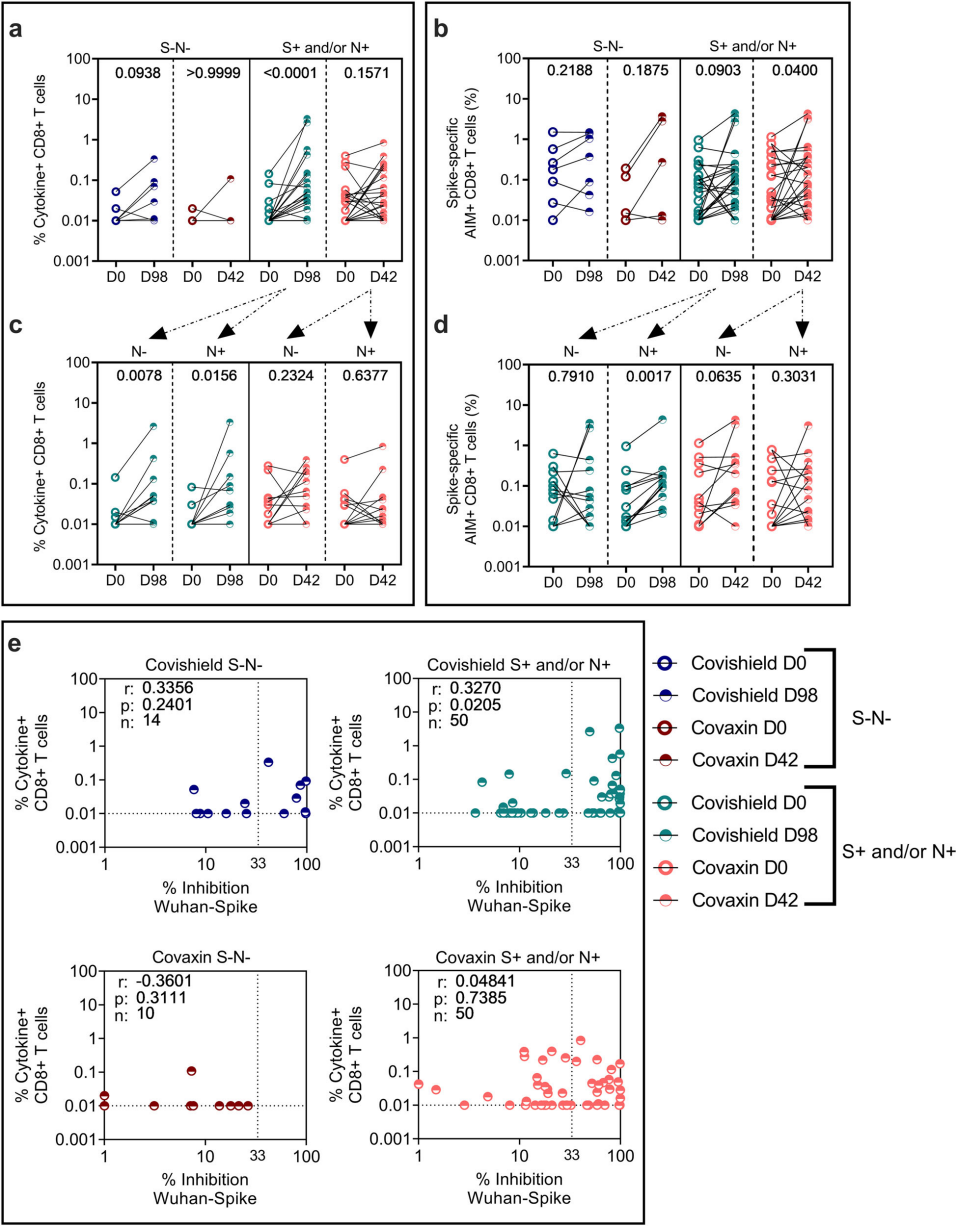
Vaccine-induced CD8 + T-cell responses to ancestral spike and comparative analysis of antibody and CD8 + T-cell responses in COVISHIELD^TM^ and COVAXIN® vaccinees. Whole Blood from COVISHIELD^TM^ and COVAXIN® individuals at baseline (D0) and 2 weeks post-vaccination (D98 for COVISHIELD^TM^ and D42 for COVAXIN®) were stimulated with spike peptide pool (1 µg/ml) for 20 h. CD8 + T-cells were analyzed for **a** intracellular expression of total cytokine+ cells (IFN-γ or IL-2 or TNF-a) obtained from Boolean gating in FlowJo and **b** for the frequencies of AIM expressing subsets (CD69 + CD137 + ). Participants were classified as either S-N- [*n* = 7 for COVISHIELD^TM^ (dark blue); *n* = 5 for COVAXIN® (dark red)] or S+ and/or N+ [*n* = 25 for COVISHIELD^TM^ (teal), *n* = 25 for COVAXIN® (pink)] at baseline. CD8 + T-cells expressing **c** effector cytokines and **d** AIM markers in subjects from the S+ and/or N+ group are further segregated as N- (*n* = 12 for COVISHIELD^TM^ and *n* = 11 for COVAXIN®) and N+ (*n* = 13 for COVISHIELD^TM^ and *n* = 14 for COVAXIN®). Line graphs represent background subtracted CD8 + T-cell frequencies. Statistical analyses was performed using a paired Wilcoxon test. **e** Correlations between total cytokine+ CD8 + T-cells (IFN-γ or IL-2 or TNF-a) in response to stimulation of whole blood with complete Wuhan spike peptide pool and corresponding neutralizing antibody responses (% inhibition) against SARS-CoV-2 Wuhan Spike at baseline and two weeks post-vaccination. Spearman’s correlation coefficient (r) and significance values (*P*) are indicated.

Study participants received either two doses of CO at 28 days apart or two doses of CS at 3 months apart as per guidelines laid by ICMR, Govt of India^6^. The peak responses to both these vaccines were measured at 2 weeks (14 days) post the second dose (D42 for CO and D98 for CS) as previously reported^10,14,24^. Antibody (Fig. 2) and T-cell response (Figs. 3 and 4) patterns in a total of 60 S+ and/or N+ and 12 S-N- subjects post primary vaccination series is summarised (Supplementary Table 2).

Significant induction of Wu-S IgG was observed in both CS and CO seropositive vaccinees, median Ab binding levels were broadly similar but a trend for higher levels were noted in CS (163,738 BAU/ml; IQR: 17,854–291,906) versus CO vaccinees (104,262 BAU/ml; IQR: 13,692–562,103) (Fig. 2a). Similarly, a highly significant increase in nAb (*p* < 0.0001) was noted at 2 weeks post-vaccination for both vaccines relative to baseline, with only 3 and 5 seropositive donors receiving CS and CO respectively, failing to respond (Fig. 2b). However, nAb levels were noticeably higher post CS vaccination, reaching near 100% inhibition in majority of CS (median: 97.57%, range: 28.94–99.76) compared to CO vaccinees (median: 58.78%, range 11.11–99.65). Accordingly, the proportion of seropositive responders with nAb titres inducing ≥90% inhibition was 62% for CS compared to 25% for CO vaccinees (Fig. 2b). While CS vaccines enhanced binding Ab (Fig. 2a) and nAb levels (Fig. 2b) across seronegative individuals relative to matched baseline as also evidenced in the larger study^23^, this did not reach statistical significance for CO vaccinees in this study. Strikingly, only CS but not CO vaccination had the potential to induce nAb with a near-100% inhibition in S-N- seronegatives (Fig. 2b), highlighting the potency of CS to be greater than CO, consistent with our earlier study^23^.

Further segregation of seropositive subjects according to basal N binding Ab, confirmed both vaccines to be immunogenic, with rising binding antibody and nAb titres in N+ and N- subjects relative to their matched baseline (Fig. 2c, d and Supplementary Fig. 4a–d). However, certain differences were noted: the boosting of nAb was uniform across all CS vaccinees, with median nAb corresponding to 97.77% inhibition in N- and 96.73% inhibition in N+ subjects (with similar baseline median Wu-S nAb in N- and N+ subjects: 8.55% for N- vs 9.975% for N + ). In the CO arm, although the median basal Wu-S nAb was similar in N+ and N- subjects (14.13% in N- vs 16.28% in N + ), nAb was enhanced to variable extents post vaccination (median: 81.34% for N- and 58.69% for N + ), with majority of the CO vaccinees displaying an increment in nAb responses. However, dampening of nAb was observed in 3 of 4 subjects who had the highest nAb prior to vaccination (Fig. 2d and Supplementary Fig. 4c, d).

Next, intracellular cytokine staining (ICS) was used to assess the combined expression of Th1 effector cytokines IFN-γ, IL-2 and TNF-α as well as T-cell activation induced markers (AIMs) (OX40 and CD137) on CD4 + T-cells following in vitro stimulation with a pool of peptides covering the complete Wuhan Spike. Supplementary Fig. 5 shows representative gating strategy for whole blood ICS assay and Supplementary Fig. 6 shows representative FACS plots for cytokine staining in a CS and CO vaccinated donor at baseline and post-vaccination. Boolean gating was used to quantify cells producing any combination of IFN-γ, TNF-α, and IL-2. Irrespective of vaccine regimen, Wu-S-specific CD4 + T-cells were robustly induced in S+ and/or N+ groups at 2 weeks post-vaccination relative to matched baseline but not in S-N- CO vaccinees (Fig. 3a). Notably, within the seropositive group receiving either CS or CO, 70% were deemed responders showing a minimum of ≥2-fold increase over baseline in CD4 + T-cell frequencies. The median fold change over baseline was 3.86-fold in CS vaccinees (median frequency of 0.044% at D0 vs. 0.17% at D98) versus 2.3-fold in CO vaccinees (median frequency of 0.052 at D0 vs. 0.12% at D42) (Fig. 3a). This data was extended and confirmed by probing AIM-marker induction (Fig. 3b). Wu-S-specific OX40 + CD137 + CD4 + T-cells were significantly enhanced in both CS (*P* = 0.0001) and CO (*P* = 0.036) vaccinees even in donors with high AIM + CD4 + T-cells at baseline (Fig. 3b). In keeping with the Ab data (Fig. 2), the presence of basal nAb did not negate induction of vaccine-induced effector cytokine CD4 + T-cells in both CS and CO vaccinees (Fig. 3c and Supplementary 7a, b); however, CO but not CS vaccine-induced AIM responses were lower in N+ subjects (Fig. 3d).

Figure 3e confirms strong correlation between nAb and CD4+ cytokine effector frequencies highlighting concomitant induction of these two arms of the immune response with the correlation significance and r-value being consistently lower for CO versus CS (Fig. 3e); another indicator of CS being more immunogenic than CO in inducing nAb and CD4 + T-cell responses.

Both vaccines as in previous studies^9,19,23^, were less efficient in inducing Wu-S-specific IFN-γ/TNF-α/IL-2 effector CD8 + T-cells (Fig. 4a) compared to the robust induction of CD4 + T-cell effectors (Fig. 3a) with significant induction noted only in CS seropositive subjects (Fig. 4a).

The median CD8+ effector cytokine frequencies post vaccination was 0.037% (range 0.01–3.3) for CS and 0.024% (range 0.01–0.84) for CO vaccinees. 60% of CS vaccinees and 36% of CO vaccinees were deemed responders (with minimum 2-fold increase in specific CD8 + T-cell frequencies over baseline). In contrast to Th1 cytokine effector induction (Fig. 4a), CO was marginally more efficient than CS in inducing CD69 + CD137 + CD8 + T-cells in seropositive vaccinees with differences relative to baseline reaching statistical significance in CO (*P* = 0.04) but not in CS (*p* = 0.0903) vaccinees (Fig. 4b). In the S-/N- group, both vaccines induced spike-specific cytokine-positive (Fig. 4a) and AIM+ (Fig. 4b) CD8 + T-cells in most subjects tested, though this did not reach statistical significance, likely reflecting the small group size. Analysis of responses in seropositive vaccinees with and without basal N Ab highlighted that CS significantly induced cytokine-positive (Fig. 4c) and AIM+ (Fig. 4d) CD8 + T-cells in N+ subjects but response to CO were blunted (Supplementary Fig. 7c, d). Further, spike-specific CD8 + T-cell responses correlated with nAb titres only in CS but not CO seropositive vaccinees (Fig. 4e); a likely reflection of differences in two vaccine platforms to present processed antigen for CD8 + T-cell induction.

The whole virus inactivated vaccine CO would be predicted to induce an immune response to a non-Spike antigen, like N, unlike the vectored CS vaccine. In support, we observed median N IgG levels to be enhanced 37-fold in CO versus 6-fold in CS seropositive vaccinees relative to their matched baseline although this difference did not reach statistical significance (Supplementary Fig. 8a) with concomitant induction of N-specific cytokine-positive CD4 + T-cells (Supplementary Fig. 8b). Although N-specific CD8 + T-cells were significantly induced in S+ and/or N + CS vaccinees, there was no boosting of the CD8 + T-cell response following CO vaccination (Supplementary Fig. 8c). We conclude that while robust induction of N-specific Ab and CD4 + T-cells was noted in both CS and CO vaccinees, CD8 + T-cells were only boosted in CS vaccinees within S+ and/or N+ group.

### Vaccine-induced nAb and T-cell responses to SARS-CoV-2 variants of concern (VOC)

**Fig. 5:**
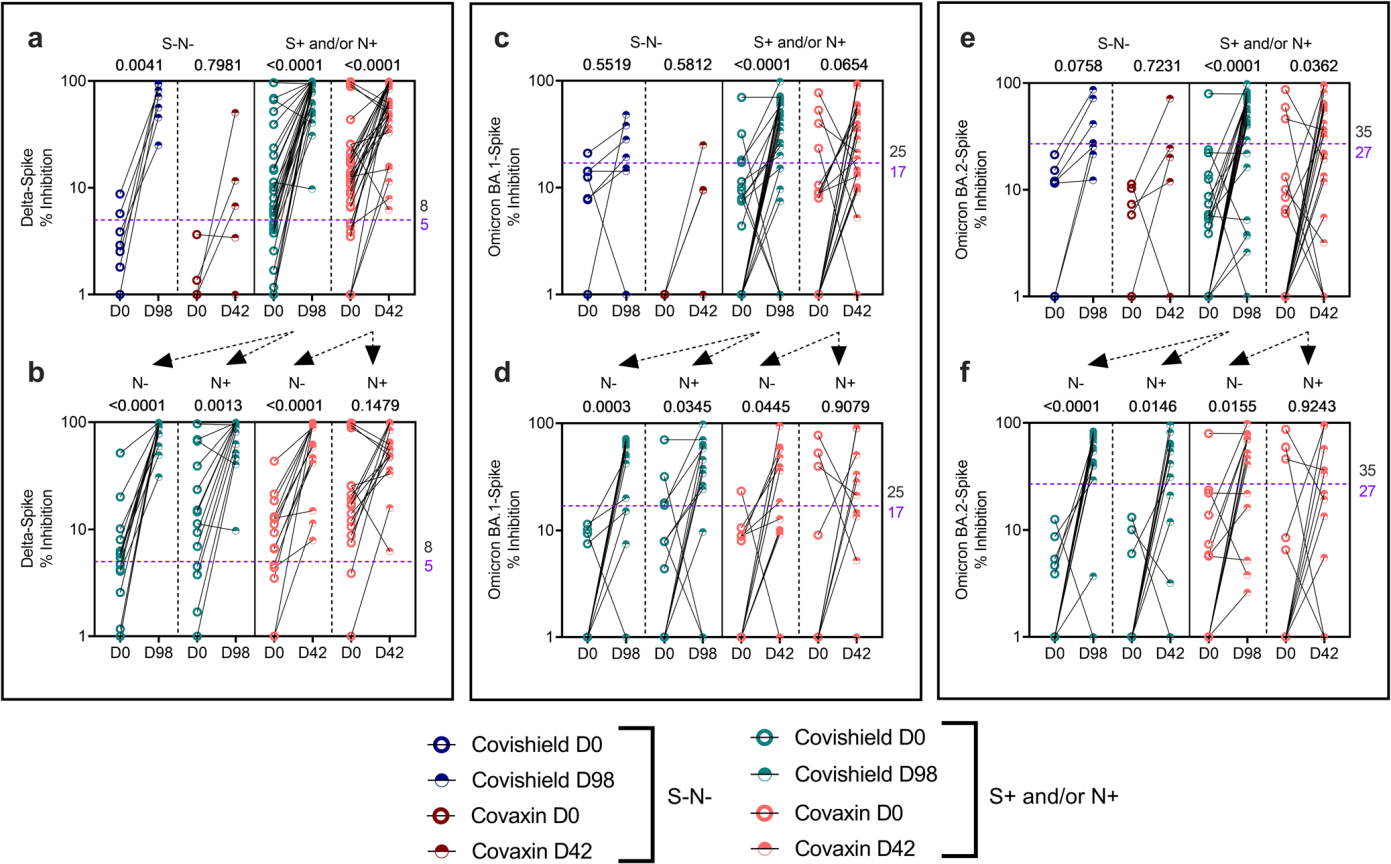
Vaccine-induced antibody responses to ancestral Wuhan and other variants of concern (VOC). Neutralizing antibody (% inhibition) against **a** Delta, **c** Omicron BA.1 and **e** BA.2 variants from S-N- [*n* = 7 for COVISHIELD^TM^ (dark blue); *n* = 5 for COVAXIN® (dark red)] and S+ and/or N+ [*n* = 29 for COVISHIELD^TM^ (teal), *n* = 31 for COVAXIN® (pink)] vaccinees measured in serum samples by MSD V-PLEX SARS-CoV-2 panel 25 (ACE2). Neutralizing antibody (% inhibition) against **b** Delta, **d** Omicron BA.1 and **f** BA.2 variants at baseline and 2 weeks post-vaccination in serum samples from the S+ and/or N+ group further segregated as N- (*n* = 15 for both COVISHIELD^TM^ and COVAXIN®) and N+ (*n* = 14 for COVISHIELD^TM^ and n = 16 for COVAXIN®). Line graphs represent antibody levels at baseline and 2 weeks post-vaccination. Assay cut-off values for each assay are denoted in purple and highest pre-pandemic cut-off in black. Two-way ANOVA with Šídák’s multiple comparisons test was used for statistical analysis.

Vaccine-induced nAb titres against Delta, BA.1 and BA.2 Omicron variants are shown in Fig. 5. The first observation is the strikingly high level of basal nAb to Delta in many samples (Fig. 5a) compared to Wu-S (Fig. 2a) consistent with more recent exposure to the Delta COVID-19 wave prior to recruitment to this study. Despite high levels, Delta nAb is significantly boosted to greater than 75% inhibition titre in both CS and CO seropositive vaccinees, with only 3 CO vaccinees with a high basal Delta nAb titre, not responding (Fig. 5a). Probing the impact of basal N Ab showed no interference in induction of Delta nAb following CS vaccination whereas it was dampened in N+ subjects receiving CO; a difference that may partly be due to pre-existing higher basal Delta nAb in N+ subjects recruited to receive CO than CS (median: 17.12% in CO versus 12.96% in CS) (Fig. 5b and Supplementary Fig. 4e, f).

Broadly the same pattern of nAb induction was noted against BA.1 (Fig. 5c, d) and BA.2 (Fig. 5e, f). Some differences included: overall titres of nAb to BA.1 and BA.2 were markedly lower compared to Wu-S and Delta. The median nAb titres in CS vaccinees to Wu-S was 97.57% (Fig. 2b), Delta 94.18% (Fig. 5a), BA.1 45.82% (Fig. 5c) and BA.2 54.34% (Fig. 5e); in CO vaccinees median nAb titres to Wu-S were 58.78% (Fig. 2b), Delta 56.54% (Fig. 5a), BA.1 13.48 % (Fig. 5c) and BA.2 19.32% (Fig. 5e). This would be consistent with Wu-S specific vaccines eliciting more restricted vaccine-specific and Delta variant specific responses since responses to Omicron variants was relatively low^20,21,25^. Nevertheless, both vaccines possessed the potential to increase BA.1 and BA.2 titres, with differences post versus pre-vaccination reaching significance or near significance (Fig. 5c, e). Interestingly, compared to N- vaccinees, CS tended to induce lower nAb to both BA.1 and BA.2, while CO failed to induce a significant increase in nAb to Omicron variants in N+ vaccinees (Fig. 5d, f, Supplementary Fig. 4g–j), highlighting that ongoing infection and/recent exposure to a circulating variant can potentially dampen rather than enhance vaccine immunogenicity reflective of potential antigenic competition.

**Fig. 6:**
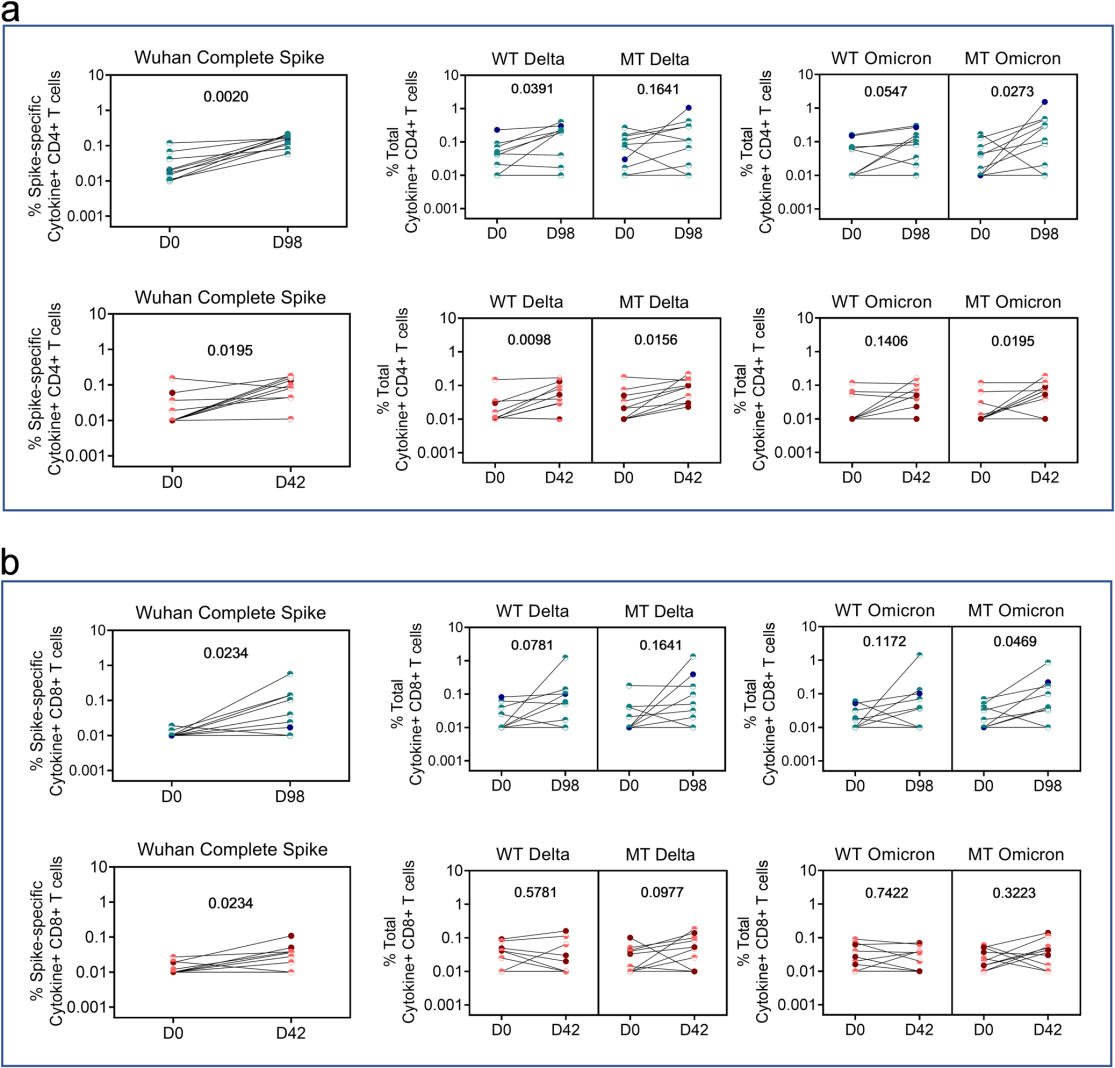
Vaccine-induced T-cell responses to ancestral Wuhan and other variants of concern (VOC). PBMCs from COVISHIELD^TM^ (*n* = 10) and COVAXIN® (*n* = 10) vaccinees at baseline (D0) and 2 weeks post-vaccination (D98 for COVISHIELD^TM^ and D42 for COVAXIN®) were either stimulated with complete spike, mutant Delta or BA.1 and BA.2 Omicron and their matched wild-type (WT) reference peptide pools (1 µg/ml) for 20 h. **a** CD4+ and **b** CD8 + T-cells were analyzed for intracellular expression of total cytokine+ cells (IFN-γ or IL-2 or TNF-a) obtained from Boolean gating in FlowJo. Line graphs represent background subtracted CD4+ and CD8 + T-cell frequencies expressing effector cytokines. Statistical analyses were performed using a paired Wilcoxon test.

The WB-ICS was initially used as a rapid screen of vaccine T-cell immunogenicity (Figs. 3 and 4). To track T-cells to VOC, we conducted ICS staining on archived PBMC on a smaller sample subset having first verified strong positive correlation between the WB and the PBMC-ICS assays conducted on matched samples to Wu-S peptide stimulation (Supplementary Fig. 9) with representative gating strategy, FACS plots shown in Supplementary Fig. 10 and 11 respectively. Effector cytokine T-cell responses to Delta and Omicron (both BA.1 and BA.2) mutant epitopes and to a peptide pool covering the entire Wu-S amino acid sequence (referred to as Wuhan complete Spike) are shown in Fig. 6. As a more specific control for the Delta and Omicron BA.1/BA.2 mutant peptides, we tested same length reference peptides (referred to as wild type: WT) that included epitopes in Wu-S spanning the precise regions covering Delta and BA.1/BA.2 mutations. Consistent with the WB-ICS data in Figs. 3 and 4, we first demonstrate both CS and CO to significantly induce CD4+ (Fig. 6a) and CD8+ (Fig. 6b) T-cells to Wu-S with the magnitude of the induced effector response being higher in CS compared to CO vaccinees. The same pattern was also noted to WT peptides though the magnitude was lower consistent with the complete Spike having greater coverage and expressing more T-cell epitopes than the WT peptides. Both vaccines enhanced CD4 + T-cell frequencies to Delta peptide with CO vaccination showing significant induction. Strikingly, we observed significant expansion of both BA.1/BA.2 specific CD4 + T-cells following vaccination with either CS or CO (Fig. 6a) which correlated with rising nAb titres (Supplementary Fig. 12a, b). Delta and Omicron BA.1/ BA.2-specific CD8 + T-cells were also expanded across some but not all donors receiving either CS or CO vaccine with differences relative to baseline reaching significance only in CS vaccinees to Omicron BA.1/BA.2 (Fig. 6b).

### Unbiased analysis of flow cytometry data extends and confirms a more robust vaccine-induced spike-specific memory CD4+ compared to CD8 + T-cell response

**Fig. 7:**
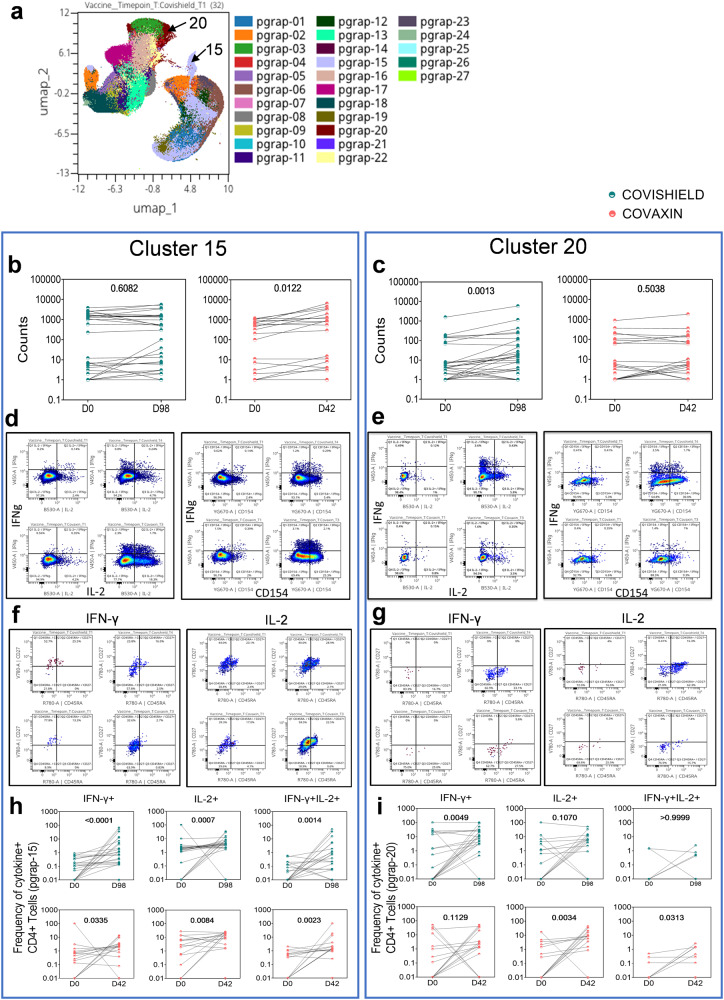
Quality of vaccine-induced spike specific CD4 + T-cell responses to Wuhan spike using OMIQ. Vaccine-induced CD4 + T-cell responses in vaccinated subjects were measured at baseline (D0) and 2 weeks post-vaccination (D98 for COVISHIELD^TM^) and (D42 for COVAXIN®). **a** Phenograph clusters expressed by CD4 + T-cells overlaid onto UMAP. **b**, **c** Pairwise comparison of numbers of cells expressed within these unique clusters pre- and post-vaccination. **d**, **e** Bivariate dot plot showing expression of IFN-γ, IL-2 and CD154 in Cluster 15 and Cluster 20 in COVISHIELD™ and COVAXIN® vaccinees. **f**, **g** Bivariate dot plot showing expression of IFN-γ+ and IL-2+ cells within different memory subsets in Cluster 15 and Cluster 20 in COVISHIELD™ and COVAXIN® vaccinees. **h**, **i** Pairwise comparison of numbers of cytokine-positive CD4+ cells expressed within these unique clusters pre and post-vaccination. Statistical analyses was performed using a paired Wilcoxon test.

Using OMIQ (http://omiq.ai) we confirmed and extended the above-described analysis of flow cytometry data. Whereas SPICE enables identification of all combinations of vaccine-induced T-cell subsets, including low frequency subsets, OMIQ software which uses dimensionality reduction enables identification of dominant vaccine-induced subsets. UMAP analysis of spike-peptide stimulated samples from 32 CS and 29 CO vaccinees highlighted the emergence of vaccine-induced T-cell clusters that was either absent or minimally expressed at baseline. Phenograph overlaid onto UMAP identified two unique clusters (pgrap15 and 20) out of a total of 27 (Fig. 7a). A Wilcoxon matched-pair signed-rank test confirmed Cluster 15 and Cluster 20 to be significantly induced in CO and CS vaccinees respectively (Fig. 7b, c). Dot plots to deconvolute the cellular subset composition identified Cluster 15 to comprise of cells that dominantly express IL-2 and AIM marker CD154 with some IFN-γ, whereas cluster 20 consisted of IFN-γ+ and CD154+ cells with low IL-2 expression (Fig. 7d, e). Gating for memory markers on total CD4 + T-cells confirmed predicted expression of both effector memory (EM) and central memory (CM) cells (Supplementary Fig. 5). However, within antigen-specific cytokine-positive cells, whereas cluster 15 included CM and EM, cluster 20 predominantly included TEMRA (effector memory T-cells re-expressing CD45RA) and EM consistent with the dominant cytokines expressed by these individual clusters (IL-2 in cluster 15 and IFN-γ in cluster 20) (Fig. 7f, g). Pairwise comparison of these cytokine-positive cells revealed IFN-γ + , IL-2+ and IFN-γ + IL-2+ cells in cluster 15 to be significantly enhanced in both CS and CO vaccinees, whereas a similar analysis of cluster 20 showed IFN-γ+ and IL-2+ cells to be dominant in CS and CO vaccinees respectively (Fig. 7h, i).

**Fig. 8:**
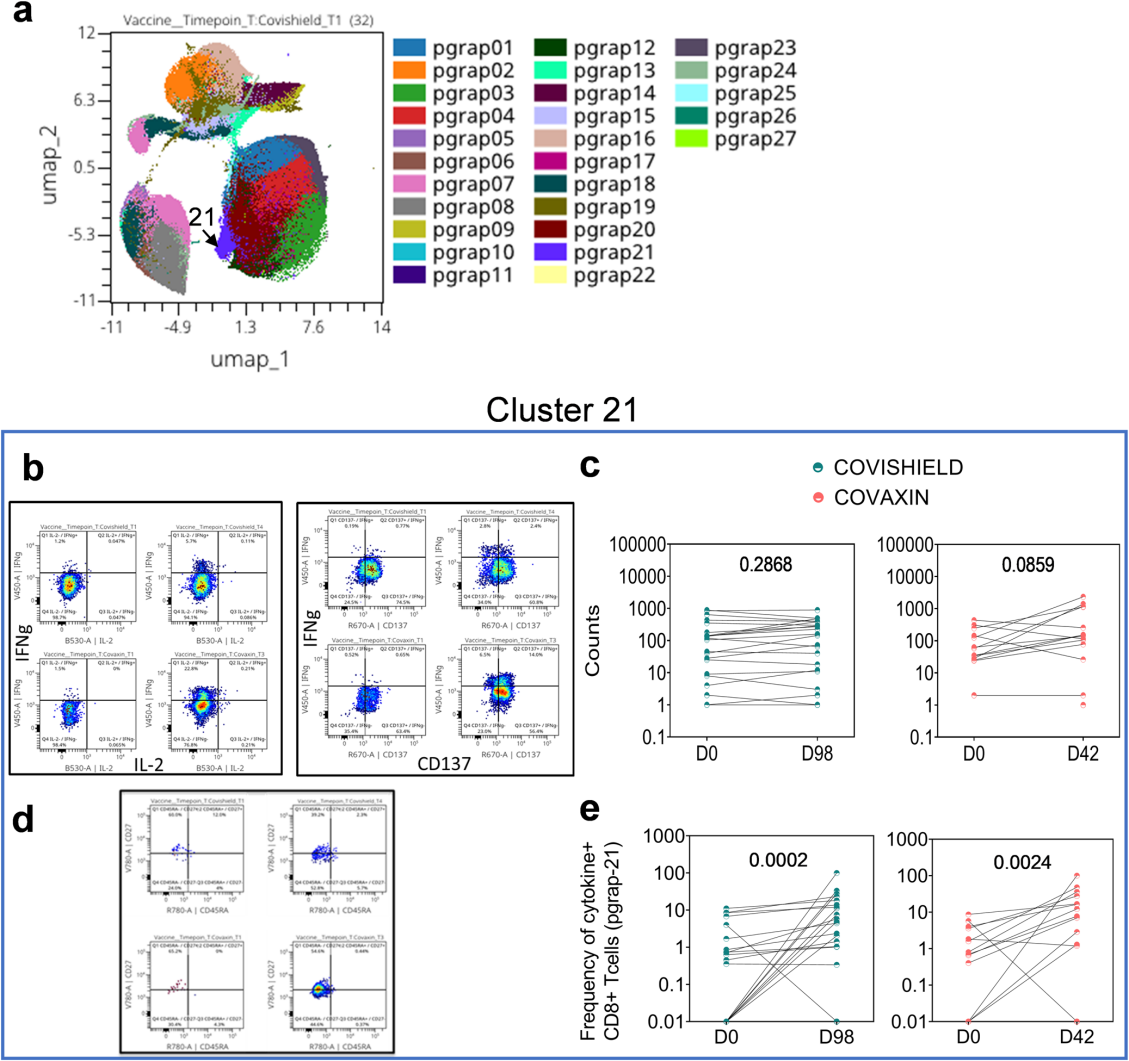
Quality of vaccine-induced spike specific CD8 + T-cell responses to Wuhan spike using OMIQ. Vaccine-induced CD8 + T-cell responses in vaccinated subjects were measured at baseline (D0) and 2 weeks post-vaccination (D98) and (D42). **a** Phenograph clusters expressed by CD8 + T-cells overlaid onto UMAP. **b** Bivariate dot plot showing expression of IFN-γ, IL-2 and CD137 in Cluster 21 in COVISHIELD™ and COVAXIN® vaccinees. **c** Pairwise comparison of numbers of cells expressed within the unique clusters pre- and post- vaccination. **d** Bivariate dot plot showing expression of IFN-γ+ cells within different memory subsets in Cluster 21 in COVISHIELD™ and COVAXIN® vaccinees. **e** Pairwise comparison of numbers of cytokine-positive CD8 + T-cells expressed within the unique clusters pre- and post-vaccination. Statistical analyses was performed using a paired Wilcoxon test.

Within the CD8 + T-cell compartment, one (pgrap21) out of a total of 27 clusters was identified, comprising predominantly cells expressing IFN-γ and CD137, although this cluster was not significantly induced by either vaccine (Fig. 8a–c) confirming both vaccines to be less potent in inducing spike-specific CD8+ compared to CD4 + T-cells. Gating for memory markers on total CD8 + T-cells identified a mixture of TEMRA, EM and CM cells, as anticipated (Supplementary Fig. 5); however, expression within vaccine-induced spike-specific cytokine-positive cells highlighted this population to be largely CM and EM (Fig. 8d, e). Taken together, this data reveals differential combinations of dominant spike-specific CD4 + T-cell cytokine effectors to be induced by CO and CS.

### Persistence of spike-specific immunity

**Fig. 9:**
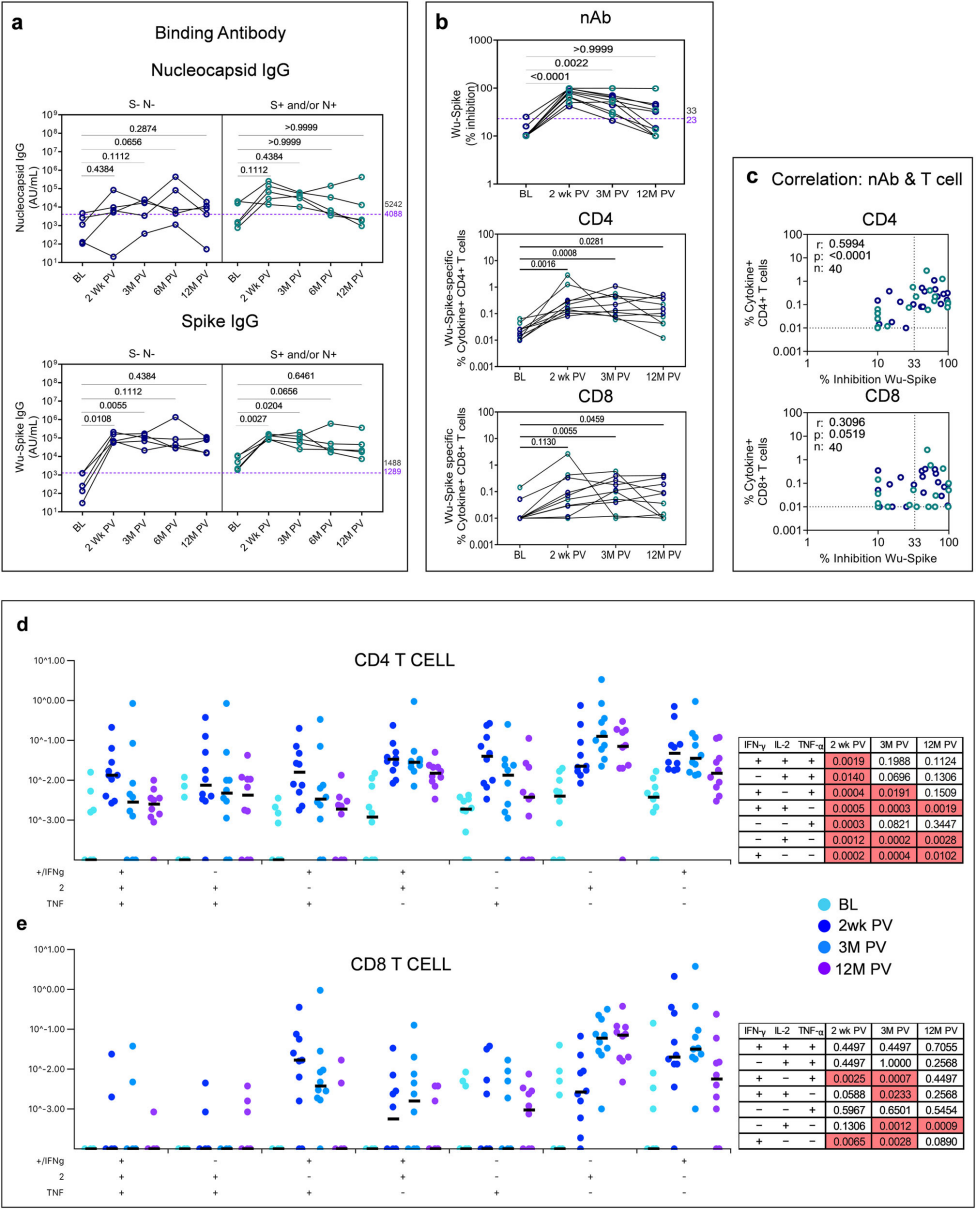
Persistence of vaccine-induced neutralizing antibodies and T-cell responses in COVISHIELD^TM^ (*n* = 10) vaccinees. **a** Antibody IgG levels to nucleocapsid and spike protein of SARS-CoV-2 were measured at baseline (BL), 2 weeks (Wk), 3-, 6- and 12-months (M) post-vaccination (PV) in serum samples by MSD V-PLEX COVID-19 Coronavirus Panel 1 (IgG). **b** Neutralizing antibody (% inhibition) against ancestral spike protein measured at baseline (BL), 2 weeks (Wk), 3- and 12-months (M) post-vaccination (PV) in serum samples by MSD V-PLEX SARS-CoV-2 panel 25 (ACE2). Assay cut-off value is denoted in purple and highest pre-pandemic cut-off in black. Whole Blood collected at baseline (BL), 2 weeks (wk), 3- and 12-months (M) post-vaccination were stimulated with spike peptide pool (1 µg/ml) for 20 hr. CD4+ and CD8 + T-cells were analyzed for intracellular expression of total cytokine+ cells (IFN-γ or IL-2 or TNF-α) obtained from Boolean gating in FlowJo. One-way ANOVA and Friedman test with Dunn’s multiple comparisons test was used. **c** Correlations between total cytokine+ T-cells and corresponding neutralizing antibody responses against SARS-CoV-2 Wuhan Spike. Spearman’s correlation coefficient (r) and significance values (*P*) are indicated. Longitudinal multifunctional spike-specific **d** CD4+ and **e** CD8 + T-cells in vaccinees. Boolean gates were created from the individual cytokines (IFN-γ, IL-2, TNF-α) in FlowJo to divide responding cells into 7 distinct subsets corresponding to all possible combinations of these functions. The data was subsequently plotted using SPICE software. Statistical significance was analyzed using Wilcoxon signed-rank sum test. Background subtracted and log data analyzed in all cases. P < 0.05 was considered statistically significant.

**Fig. 10:**
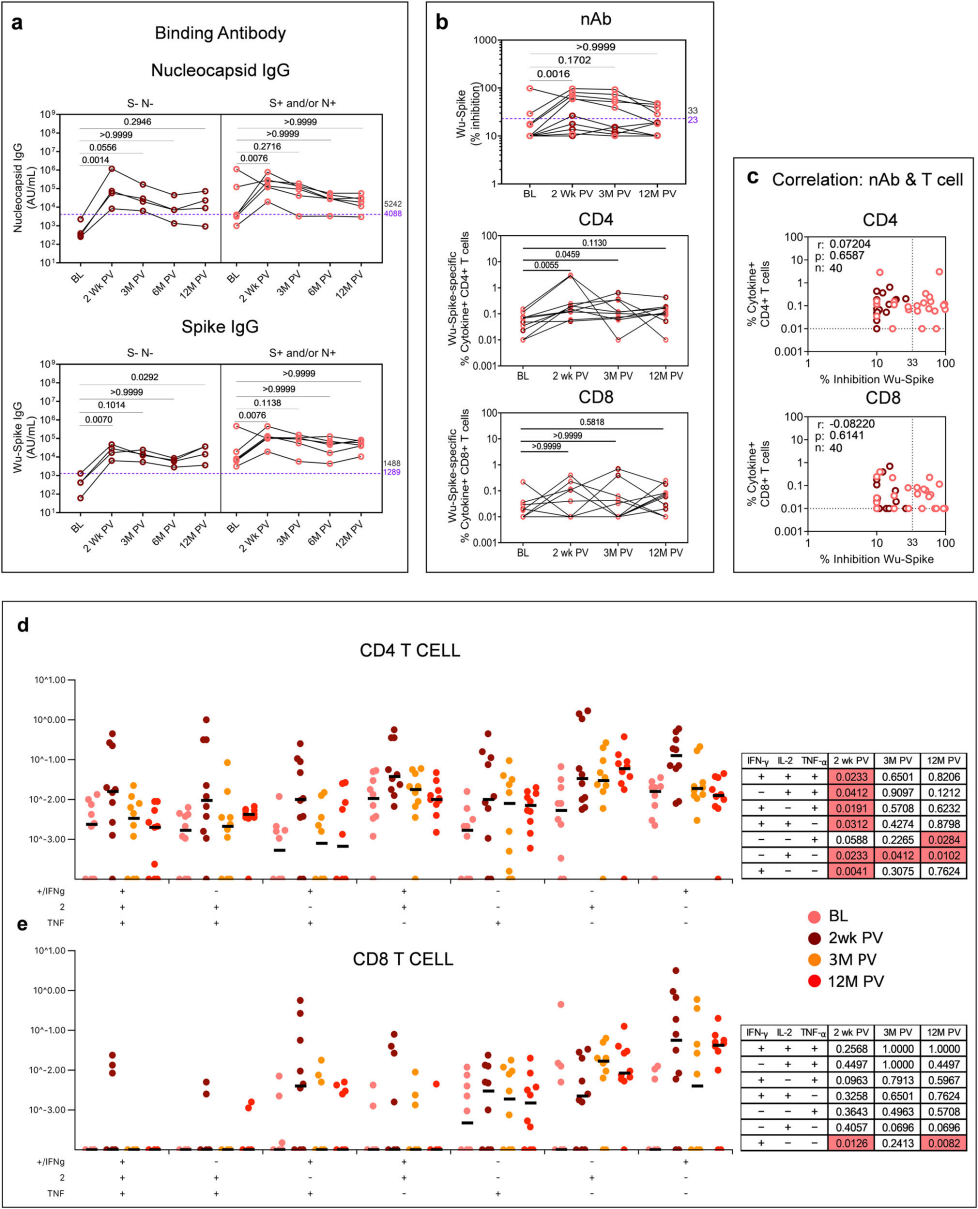
Persistence of vaccine-induced neutralizing antibodies and T-cell responses in COVAXIN® vaccinees (*n* = 10). **a** Antibody IgG levels to nucleocapsid and spike protein of SARS-CoV-2 were measured at baseline (BL), 2 weeks (Wk), 3-, 6- and 12-months (M) post-vaccination (PV) in serum samples by MSD V-PLEX COVID-19 Coronavirus Panel 1 (IgG). **b** Neutralizing antibody (% inhibition) against ancestral spike protein measured at baseline (BL), 2 weeks (Wk), 3- and 12-months (M) post-vaccination (PV) in serum samples by MSD V-PLEX SARS-CoV-2 panel 25 (ACE2). Assay cut-off value is denoted in purple and highest pre-pandemic cut-off in black. Whole Blood collected at baseline (BL), 2 weeks (wk), 3- and 12-months (M) post-vaccination were stimulated with spike peptide pool (1ug/ml) for 20 h. CD4+ and CD8 + T-cells were analyzed for intracellular expression of total cytokine+ cells (IFN-γ or IL-2 or TNF-α) obtained from Boolean gating in FlowJo. One-way ANOVA and Friedman test with Dunn’s multiple comparisons test was used. **c** Correlations between total cytokine+ T-cells and corresponding neutralizing antibody responses against SARS-CoV-2 Wuhan Spike. Spearman’s correlation coefficient (r) and significance values (*P*) are indicated. Longitudinal multifunctional spike-specific **d** CD4+ and **e** CD8 + T-cells in vaccinees. Boolean gates were created from the individual cytokines (IFN-γ, IL-2, TNF-α) in FlowJo to divide responding cells into 7 distinct subsets corresponding to all possible combinations of these functions. The data was subsequently plotted using SPICE software. Statistical significance was analyzed using Wilcoxon signed-rank sum test. Background subtracted and log data analyzed in all cases. *P* < 0.05 was considered statistically significant.

Persistence of vaccine-induced immunity was assessed in ten samples collected to date by extending the analysis beyond two weeks (Figs. 1–8) to 3 and 12 months post primary vaccination (Figs. 9 and 10). A striking feature of these latter time points was evidence of rising N and S binding antibodies across all samples bar one, including the N-S- samples at baseline (Figs. 9a and 10a), consistent with widespread exposure during the Omicron wave in India when these samples were collected. Immunity to ancestral Spike in these ten S + N+ samples showed maintenance of Wu-Spike specific nAb in CS vaccinees until 12 months with a decline noted in some subjects beyond 3 months (Fig. 9b). CD4+ and CD8 + T-cells also persisted until 12 months with the magnitude of the CD4 + T-cell response being consistently higher (Fig. 9b), confirmed by strong correlation between nAb and CD4+ but weak correlation between nAb and CD8 + T-cell responses (Fig. 9c). CO vaccinees, however, segregated into two distinct groups: responders, who were nAb positive at 2 weeks showed persistent nAb at 12 months and non-responders who remained nAb low/negative throughout, indicating non-responders do not necessarily convert to responders with time (Fig. 10b). While spike-specific CD4 + T-cell responses persisted in some but not all CO vaccinees, CD8 + T-cell responses were not significantly induced over baseline (Fig. 10b) and both CD4+ and CD8 + T-cell responses showed poor correlation with nAb (Fig. 10c).

Several SARS-CoV-2 vaccines have been shown to induce multifunctional T-cells, implicated to distinguish high versus low vaccine responders^26–28^. Polyfunctionality was assessed in T-cells induced to express 3, 2, and 1 (7 total) combinations of the three major Th1 effector cytokines: IFN-γ, IL-2, and TNF-α using SPICE software^29^ relative to matched baseline over time. Significant induction of cell subsets is highlighted (pink boxes Figs. 9 and 10). In CS vaccinees polyfunctional CD4 + T-cells peaked at 2 weeks, then gradually declined (Fig. 9d), but polyfunctional CD8 + T-cells peaked later at 3 months post vaccine (Fig. 9e). In CO vaccinees, polyfunctional CD4 + T-cells were clearly evident at 2 weeks post vaccine (Fig. 10d) but polyfunctional CD8 + T-cells were not observed in concurrence with the weak induction of CD8 + T cell responses following CO vaccination (Fig. 10e).

Taken together, the data demonstrate both vaccines to primarily elicit a strong Th1—biased polyfunctional CD4 + T-cell response and a weaker CD8 + T-cell response with absence of polyfunctional CD8 + T-cells noted especially in CO vaccinees.

## Discussion

Understanding the immunogenicity of COVID-19 vaccines in seropositive subjects with varying basal levels of SARS-CoV-2 specific binding antibodies, neutralising antibodies and T-cell responses is central to assessing the capacity of a vaccine to enhance pre-existing immunity. This manuscript addresses this important question.

CS and CO significantly boosted circulating Ab binding levels, nAb titres, effector CD4 + T-cell responses and AIM + CD4 + T-cells. SARS-CoV-2 specific CD8 + T-cell responses were less potently induced; however, we report that AIM + CD8 + T-cells and single IFN-γ + CD8 + T-cells were significantly expanded by both vaccines. Importantly, dimensionality reduction and OMIQ analysis of flow cytometry data revealed diversification of the vaccine-induced T-cell response with the emergence of unique cytokine-positive clusters within antigen-specific cells that were absent at baseline. CO boosted predominantly IL-2 + CM and EM cells, whereas CS boosted predominantly IFN-γ + EM and TEMRA CD4 + T-cells. CD4 + TEMRA cells have been previously implicated in protective immunity against pathogens constituting a preformed effector population, noted in COVID-19 infection^30^ as well as AZD1222 vaccination^31^. CS was more efficient than CO in expanding IFN-γ + CM and EM CD8 + T-cell subsets. Beyond boosting the T-cell magnitude, both vaccines broadened the immune response by boosting Ab and T-cell responses to the Delta variant in particular, even in subjects with high basal Delta nAb titres, and to a lesser degree to the Omicron BA.1 and BA.2 variants, in keeping with publications demonstrating Wuhan-Spike encoding vaccines to be highly cross-immunogenic for Delta but, to a lesser extent, Omicron strains^18–21,25,32,33^. It would be interesting to probe if CS and CO induced immune responses target SARS-CoV-2 strains that emerged post BA.1 and BA.2 as part of our planned future studies when sample collection is completed, especially as other reports show significant immune escape in Omicron sub-lineages: BQ.1.1, XBB, XBB.1, and XBB.1.5^34^.

Our data provide unequivocal evidence that pre-existing SARS-CoV-2 specific immunity does not abrogate CS and CO immunogenicity consistent with published reports. Thus, immunogenicity studies with CS in seropositive Indian healthcare workers and Bangladeshi adults demonstrated a dramatic increase in RBD binding antibody titres that was maintained for 3–4 months following two vaccine doses^7,35^. Moreover, nAb levels were enhanced ~40-fold post-prime in CS vaccinees^36^. ChAdOx1nCoV-19 immunization induced antibodies in UK and Brazil cohorts having a history of infection compared to vaccine-naïve individuals^37,38^. Enhanced T-cell responses have been noted in vaccinated seropositive individuals including predominant IFN-γ expression by ELISPOT and ICS assays^36^. In addition, single-dose vaccination elicited higher memory T- and B-cell responses in SARS-CoV-2 infected individuals compared to those without prior exposure^39^. The larger study^23^ included a higher number of TrimericS seronegative recruits and showed CS induced robust antibody responses specific to Wuhan, Delta, and Omicron, whereas CO responses were poor.

Beyond enhancing magnitude, we provide data on the capacity of CS and CO to enhance the quality of pre-existing T-cell immunity in seropositive subjects. Polyfunctional T-cells are of recognised importance in antiviral immunity^40^, and we^28^ and others^9,27^ have previously demonstrated that AZD1222/CS induced such cells in seronegative subjects. A noticeable difference in this study is the observation of pre-existing polyfunctional CD4 + T-cells at baseline, including 3+ cells expressing IFN-γ, IL-2 and TNF-α. Given that seropositive subjects recruited had not been previously vaccinated, this data highlights that infection/exposure alone has the capacity to induce spike-specific polyfunctional CD4 + T-cells beyond Ab responses. Although the protective efficacy of a Th1-skewed CD4+ or CD8 + T-cell response in reducing clinical severity has been highlighted by several clinical trials, direct evidence in protection is unclear. It is assumed that both subsets play a conventional role: with SARS-CoV-2 specific CD4 + T-cells promoting nAb and CD8 + T-cells playing a direct effector role in viral control and clearance^41^. Studies conducted prior to the global COVID-19 vaccine rollout demonstrated infection to drive significant expansion of spike-specific nAb, CD4+ and CD8 + T-cell responses^42^ and to be noticeably of greater magnitude than responses induced in seronegative unexposed subjects by CS or CO^16^ or indeed mRNA vaccines^41^ indicating primary immune responses induced by vaccination to be inherently of lower magnitude than that induced by infection. There is also consensus for both the vectored and inactivated vaccines to be less potent in inducing CD8 + T-cell responses^9,19,27^, linked to putative differential processing and presentation on antigen presenting cells^43,44^. SARS-CoV-2 spike engineered into an adenoviral vector (CS) has the potential to induce both CD4+ and CD8 + T-cell responses linked to processing through both the endogenous and exogenous pathways^43^, supported by our OMIQ analysis identifying the dominant induction of IFN-γ + EM CD4+ and CD8 + T-cells in CS vaccinees. In contrast, the whole virus inactivated CO vaccine is likely processed and presented by dendritic cells (DCs) via the MHC class II pathway^44^, supported also by OMIQ analysis identifying the dominant induction of IL-2 + CM, EM CD4 + T-cells in CO vaccinees. Although adjuvants in the inactivated SARS-CoV-2 vaccine may enhance cross-presentation of viral antigen to CD8 + T-cells by DCs, the magnitude of the CD8 + T-cell immune response is likely to be lower compared to that induced by prolonged antigenic expression by a vectored vaccine that facilitates the maintenance of effector CD8 + T-cells^44^.

Taken together this manuscript provides unequivocal data to confirm both CS and CO vaccines to be immunogenic in subjects pre-exposed to the COVID-19 pandemic in India with clear evidence of the capacity of these vaccines to broaden and enhance the quality of SARS-CoV-2-specific immunity. Substantial evidence reinforces the importance and benefit of vaccination, despite previous infection, in imparting protection against infection and severe disease^45,46^. Additionally, several observational studies and randomized immunogenicity trials have emphasized that the combination of vaccination and infection (hybrid immunity) can significantly enhance the magnitude and durability of protection compared to infection alone^47–49^, with mRNA vaccines being highlighted to be superior than the vectored AZD1222 vaccine^50^. Furthermore, heterologous prime-boost strategies have proven to be superior compared to homologous boosting^51^. Pertinently, Chaudhary et al. recently demonstrated that boosting with CS after CO primary series induced substantial increase in spike-specific neutralizing antibodies and T-cell responses^52^.

We provide fresh insight showing vaccine-induced immune responses in subjects with recent / ongoing infection reflected by the presence of circulating nucleocapsid antibody to be dampened rather than enhanced compared to subjects without N Ab. This is likely due to antigenic competition and has only been seen in CO vaccinees, possibly due to the broader array of SARS-CoV-2 antigens expressed by CO compared to CS vaccine^8,53^. Moreover, inter-vaccination gap is necessary to avoid antigenic competition which can substantially reduce vaccine efficacy^54,55^, highlighting timing of vaccination needs to be considered especially for subjects with evidence of ongoing / recent infection.

The major outcome of this study is that the first two COVID-19 vaccines rolled out by the Govt. of India are both immunogenic in a pre-exposed seropositive population receiving their primary vaccination, which strongly argues against vaccine hesitancy; specifically, the argument that natural exposure per se provides adequate protection and/or cannot be further enhanced^56,57^. This is especially important as both COVISHIELD^TM^ produced by the Serum Institute of India and COVAXIN® by BBIL have been globally distributed; our study is therefore pertinent to significant parts of the global population which is yet to receive their primary vaccination. The second major outcome of our study is that it highlights diversity in the quality of T-cell responses: between vaccines and between the CD4+ and CD8 + T-cell compartments; given the wider importance of T-cell quality beyond magnitude being important in antiviral immunity^26,27^, our data calls for further studies to understand the functional nature of SARS-CoV-2 -specific T-cell immune responses and their persistence in the context of both infection and vaccination. Combining knowledge of both T-cell immunity and nAb responses will be essential in planning for future SARS-CoV-2 vaccine strategy in the context of vaccine interval and booster doses.”

## Methods

### Study design and participants

This study was performed in healthy adults aged 18–44 years at St. John’s Research Institute. The trial was approved by the Institutional Ethics Committee (SJRI-298/2021) and registered at the Clinical Trial Registry of India (CTRI) website (CTRI/2021/09/036258). All participants provided written informed consent prior to enrolment. Written informed consent was obtained from all individuals according to the Declaration of Helsinki. SARS-CoV-2 vaccine-naïve participants were screened for serostatus using a combination of anti-spike and/or anti-nucleocapsid antibodies, either qualitative or quantitative, manufactured by either Roche Diagnostics, Abbott Laboratories or Liaison DiaSorin. Recruitment was done using a combination of unbiased as well serostatus-confirmed inclusion. Baseline samples were re-tested and classified for their serostatus using DiaSorin TrimericS and MSD platforms at Christian Medical College, Vellore (see below). Participants with a history of medical illness or prior severe COVID-19 that required ventilation or administration of biologics such as convalescent plasma or monoclonal antibodies were excluded. Vaccine-naïve participants were administered two doses of either COVAXIN® at 4-week interval or COVISHIELD^TM^ at 12-week interval as per the prevailing government norms. Allocation of participants to vaccine arms was non-randomized and per participant-choice. Participants in the COVAXIN® arm were sampled at Day 0 (prior to first dose of vaccine) and Day 42 (2 weeks post-vaccination). Participants in the COVISHIELD^TM^ arm were sampled at Day 0 (prior to the vaccine dose) and Day 98 (2 weeks post-vaccination). Participants were followed bi-monthly for symptoms of COVID-like illness. A consort flow chart showing the details of the recruitment and study protocol has been provided in Supplementary Fig. 1. The median age of the study population was 28 years (range, 18–44 years), and 65% were male and 35% were female subjects. The details of the study participants were included in Supplementary File 1.

### Anti-spike IgG measured using the LIAISON® SARS-CoV2 TrimericS IgG assay

Binding antibodies against the spike protein in all study participants were measured using the LIAISON® SARS-CoV-2 TrimericS IgG assay on the DiaSorin platform according to the manufacturer’s instructions, as described previously^23,58^. The LIAISON® SARS-CoV-2 TrimericS IgG assay, an indirect chemiluminescence immunoassay, was used for the quantitative determination of specific IgG antibodies including neutralizing antibodies against the SARS-CoV-2 trimeric spike glycoprotein in human serum samples. The Trimeric protein is a stabilized native form of the spike that improves the detection of neutralizing antibodies. Briefly, all serum samples were inactivated at 56 °C for 30 min before being tested. The assay has a range of 4.81 to 2080 BAU/ml (Binding Antibody Units/ml). Samples with high titres (>2080 BAU/ml) were diluted further as per the kit manufacturer’s guidelines. The binding antibody units measured in this assay are mapped to the 1st WHO international standard for anti-SARS-CoV-2 immunoglobulin (NIBSC Code-20/136). The assay cut-off of 33.8 BAU/ml was used to determine seropositivity. In samples with titre <33.8 BAU/ml, seroconversion was defined as an increase in titre to >33.8 BAU/ml following vaccination. In samples with titre >33.8 BAU/ml, seroconversion was defined as at least 2-fold increase in titre following vaccination.

### IgG quantification by MSD

Immunoglobulin G (IgG) antibodies against SARS-CoV-2 spike and nucleocapsid proteins were measured using an MSD V-PLEX COVID-19 Coronavirus Panel 2 (K15369) kit. Multiplex Meso Scale Discovery electrochemiluminescence (MSD-ECL) assays were performed according to the manufacturer’s instructions as described previously^23,59^. All serum samples were inactivated at 56 °C for 30 min before being tested. Briefly, 96-well plates were blocked at room temperature for at least 30 min. Plates were then washed; samples were diluted 1:5000 and added to the plates along with serially diluted reference standard and serology controls. Plates were incubated for 2 h and further washed. SULFO-TAG detection antibody was added, and plates were incubated for 1 h. After incubation, plates were washed and read using a MESO Sector S 600 plate reader. Data were generated by Methodological Mind software and analyzed using MSD Discovery Workbench (version 4.0). Results were normalized to standard(s) and expressed as MSD arbitrary units per mL (AU/mL).

### Quantification of ACE2 receptor blocking/neutralizing antibody

A multiplexed MSD immunoassay was used to measure the ability of vaccine-induced antibodies in heat inactivated serum to block ACE2 binding to SARS-CoV-2-S, thereby evaluating the functional potential of neutralizing antibodies to compete with the ACE2 receptor for binding to SARS-CoV-2-S. Multi-spot, 96-well, V-PLEX plates coated with SARS-CoV-2-S WT (Wuhan-Hu-1), B.1.1.7, B.1.351, B.1.617.2, and B.1.1.529; BA.1, BA.2, and BA.3, were used for the quantification of ACE2 receptor blocking [V-PLEX SARS-CoV-2 Panel 13 (ACE2) kit (K15466U-2) and Panel 25 (ACE2) kit (K15586U-2)]. The assays were performed according to the manufacturer’s protocol as described previously^23,59^. Serum or plasma samples were diluted 1:10 and 1:100 in diluent buffer. For panel 13 assays, an ACE2 calibration reagent provided by the manufacturer was added, but no calibration reagent was provided for panel 25. Plates were read on a MESO SECTOR S600 Reader. Raw data was processed by MSD Discovery Workbench Software (Version 4.0). Quantifications were reported in U/mL and percentage of ACE2 receptor blocking for panel 13 and in percentage of ACE2 receptor blocking for panel 25.

### Peptides for antigen stimulation

All peptide pools (15-mer sequences with 11 amino acids overlap) were procured from Miltenyi Biotech and the final concentration used for stimulation was 1 μg/ml. To measure responses to ancestral Wuhan, we used PepTivator SARS-CoV-2 Prot_S Complete pool that covers the whole protein coding sequence of the surface or spike glycoprotein (“S”) without the first four amino acids of the signal peptide and includes all functional domains (predicted immunodominant sequence domains), (N-terminal S1 domain) and (part of the C-terminal S2 domain). To probe T cell responses to SARS-CoV-2 VOC (Delta and Omicron BA.1 and BA.2), we used matched peptide pairs from Miltenyi, which included peptides spanning the mutation and matched reference peptides in the ancestral strain enabling head-to-head comparison of the ancestral and mutant responses. The PepTivator Mutation Pools selectively cover the mutated regions in the surface or spike glycoprotein (“S”) of the respective variant, whereas WT Reference Pools cover the homologous domains of the Wuhan sequence. Hence, the responses to the mutant VOC and WT reference peptides are comparable.

In the B.1.617.2 lineage (Delta variant), there are in total 10 mutations in the spike glycoprotein compared to the Wuhan variant (reference genome GenBank MN908947.3. The PepTivator SARS-CoV-2 Prot_S B.1.617.2 Mutation Pool selectively covers the mutated regions and consists of 32 peptides of 15 aa length. As its control, the PepTivator SARS-CoV-2 Prot_S B.1.617.2 WT Reference Pool has been used that consists of the 32 homologous peptides of the Wuhan sequence of the Wuhan variant.In the B.1.1.529/BA.1 lineage (Omicron variant), there are in total 37 mutations in the spike glycoprotein compared to the Wuhan variant. The PepTivator® SARS-CoV-2 Prot_S B.1.1.529/BA.1 Mutation Pool selectively covers the mutated regions and consists of 83 peptides of 15 aa length. For control purposes, a respective PepTivator SARS-CoV-2 Prot_S B.1.1.529/BA.1 WT Reference Pool, covering the homologous domains of the Wuhan sequence has been used. In the B.1.1.529/BA.2 lineage (Omicron variant), there are in total 31 mutations in the spike glycoprotein compared to the Wuhan variant. The PepTivator SARS-CoV-2 Prot_S B.1.1.529/BA.2 Mutation Pool selectively covers the mutated regions and consists of 68 peptides of 15 aa length. For control purposes, the respective PepTivator SARS-CoV-2 Prot_S B.1.1.529/BA.2 WT Reference Pool, covering the homologous domains of the Wuhan sequence has been used. Further details of the peptides and the sequences of the mutated regions are mentioned in the manufacturer’s website.

### Whole blood-based T-cell intracellular cytokine staining (ICS) assay set-up

Blood was collected in sodium heparin tubes and processed within 3 h of collection. The whole blood-based SARS-CoV-2-specific T-cell ICS assay was adapted from a previously reported whole blood ICS assay optimized to quantitate Mycobacterium tuberculosis-specific T-cells in small volumes of blood with minor modification^60^. Stimuli included: SARS-CoV-2 PepTivator peptide pools (Miltenyi Biotec, Woking, UK), consisting of 15mer sequences with 11-amino-acid overlap spanning the entire sequence of SARS-CoV-2 structural proteins including bespoke peptides covering spike, nucleocapsid and membrane proteins. All peptides were used at a final concentration of 1 µg/ml. Briefly, 300 µL whole blood was pipetted into 5 ml polypropylene tubes (Sarstedt, Germany) and stimulated with the SARS-CoV-2 spike, nucleocapsid and membrane protein peptide pool at 37 °C for 20 h in the presence of costimulatory antibodies against CD28 and CD49d (1 µg/mL; BD Biosciences, San Jose, CA, USA). Golgi-Plug brefeldin-A and Golgi-Stop monensin (1×, BioLegend Louis, MO, USA) were included for the last 18 h. Unstimulated blood incubated with costimulatory antibodies, no peptide, served as the negative control. CEFT peptide pool at 1 μg/ml (JPT Peptide Technologies) and Phytohemagglutinin at 2 μg/ml (PHA, Remel) were included as common recall antigen and positive control respectively. Post incubation, cultures were subjected to red blood cell lysis and white cell fixation as a single step using 1× FACS lysis buffer (BD, San Diego, CA, USA) for 10 min. The cells were washed and cryopreserved in freezing media (50% fetal bovine serum, 40% RPMI and 10% DMSO) and stored −80 deep freezer until batched analysis.

### PBMC ICS assay to track SARS-CoV-2 specific T-cell responses: Assay set-up

Blood (16–20 ml) was collected in Na-Heparin tubes (BD, Franklin Lakes NJ, USA) and peripheral blood mononuclear cells (PBMCs) were isolated using 15 ml ACCUSPIN (Sigma-Aldrich) tubes by density centrifugation as described previously. SARS-CoV-2-specific CD4+ and CD8 + T-cell responses were tracked using a validated ICS assay^28^. Briefly, cryopreserved PBMC were thawed, and seeded in 96-well round-bottom plates (Costar) at 1 × 10^6^ cells/well in complete RPMI medium {RPMI-1640 (1X) + GlutaMAX™−1 + 25 mM HEPES [Invitrogen] supplemented with 10% FCS [Thermo Fisher Scientific], 100 U/mL penicillin and 100 μg/mL streptomycin} after 2 h of rest. Cells were stimulated for 20 h at 37 °C with Peptivator peptide pools (Miltenyi Biotec) spanning the entire sequence of SARS-CoV-2 structural proteins, i.e., complete spike (S), nucleocapsid (N) or membrane (M) at a final concentration of 1 μg/ml and costimulatory antibodies CD28/49d at 1 μg/ml. In selected experiments, cells were also stimulated with the Delta variant (B.1.617.2), Omicron (B.1.1.529) BA.1 and BA.2 variants and their matched wild-type peptide pools (Miltenyi Biotec; Delta: 130–128–761 and 763, Omicron BA.1: 130–129–927 and 928 and BA.2: 130–130–806 and 807). For negative control, cells were incubated with costimulatory antibodies, no peptide. Phytohemagglutinin (PHA, Remel) 4 μg/ml was included as positive control respectively. Brefeldin A and Monensin (1×, BioLegend) were included in the last 18 h to prevent cytokine release.

### Flow cytometry: staining and acquisition

Cell staining was performed on cryopreserved sample from whole blood (WB)-ICS assay (see above) or on stimulated PBMC-ICS assay samples (see above). For WB-ICS assay, samples were thawed and washed before staining with the 17-colour panel consisting of antibodies listed in Supplementary Table 3. Staining for cell surface markers was first conducted with a cocktail of antibodies at room temperature for 30 min. Cells were then permeabilised with a 1× perm/wash buffer (BD) and stained for intracellular cytokines and AIM markers with a cocktail of antibodies at room temperature for 30 min. PBMCs were stained with a panel of antibodies (Supplementary Table 4) by a process described previously^28^.

Samples were acquired on a BD FACSAria Fusion flow cytometer and analysed using FlowJo version 10.8.1 (FlowJo, Ashland, OR, USA). A positive response was defined as minimum 2-fold above matched unstimulated cells, with an assay cut-off for total specific cytokine-positive CD4+ and CD8 + T-cells expressing dominant cytokines: IFN-γ, IL-2, and TNF-α alpha taken as 0.01. All longitudinal samples from a given donor were stained on the same day. Optimised PMT setting were pre-determined and followed throughout the study. In case of unforeseen changes in instrument set up during the course of the study due to laser/instrument failure, the following mitigating step was carried out: T-cells stimulated with SARS-CoV-2 peptides and PHA were rerun sequentially on the same day on the chosen settings and effector cytokine gates compared directly for T-cell frequencies with gates accordingly adjusted to ensure comparable frequencies on the same sample set acquired on different instrument settings. A single sample stimulated with PHA and run in parallel to each test served as an inter-assay variation control (IVC), Supplementary Fig. 13 shows IVC for whole blood ICS assay with a coefficient of variation (CV) for CD4 + T-cells being 17.41% for batch 1 and 6.67% for batch 2; for CD8 + T-cells CV was 18.1% for batch 1 and 19.53% for batch 2 giving confidence that a 2-fold increase of a test response above matched negative control accommodates the assay CV.

### Flow cytometry data analysis

Cell fluorescence was acquired on the 5-laser, 18-parameter BD FACSAria™ Fusion flow cytometer (BD Biosciences, San Jose, CA) using BD FACSDiva™ version 8.0.1 software, as previously described^28^. Samples were analyzed using FlowJo 10.8.0 (BD Biosciences). Briefly, gating sequence included the following order: FSC-H vs FSC-A to exclude doublets; singlet gate; exclusion of dead cells using a vital dye and DUMP lineage markers (see Supplementary Tables 3 and 4) on PBMC or DUMP lineage markers without live/dead stain for WB ICS; lymphocyte gate based on FSC/SSC; major lineage gates, namely, CD3, CD4, CD8 and lastly gates set for effector cytokines or AIM markers or memory subsets within the CD3,CD4 double positive or CD3,CD8 double positive subsets. Effector cytokine gates were strictly maintained across all samples. Antigen specific cytokine frequencies were calculated after background subtraction on sample matched negative control. Background subtractions were performed in Pestle version 2. Polyfunctionality of CD4+ cells and CD8+ cells expressing combinations of IFN-γ, IL-2, TNF-α was analyzed with SPICE version 6.1 software^29^ as described previously^28^. In addition, UMAP, Phenograph analysis was performed using OMIQ data analysis software (www.omiq.ai) as described previously^28^. FCS files were uploaded to OMIQ platform along with meta data information. Compensation was performed using single colour ultra-comp beads. Cells were sequentially gated into singlets based on FSC-A vs FSC-H > live cells >CD3+ and CD3-. CD3+ cells are further classified in to CD4+ and CD8+ cells. CD4+ and CD8+ cells from both groups and time points were digitally concatenated and randomly subsampled to 2 million cells where each group and time point has equal numbers of cells. UMAP and FlowSOM was performed on the subsampled CD4+ cells. Edge R was used for statistical tests; 1 and 0.6 million cells were subsampled respectively for the OMIQ analysis.

### Statistical analyses

Graphical representations were performed in Prism version 8.4.3 (GraphPad, San Diego, CA, USA). Statistical tests were performed in Prism. Nonparametric tests were used for all comparisons. One-way ANOVA with Bonferroni’s multiple comparison test, Friedman test with Dunn’s multiple comparisons test, Two-way ANOVA with Šídák’s multiple comparison test was used for multiple comparisons. Mann–Whitney and Wilcoxon matched-pairs signed rank test was used for unmatched and paired samples, respectively.

### Reporting summary

Further information on research design is available in the Nature Research Reporting Summary linked to this article.

### Supplementary information

Supplementary Figures and Tables

SUPPLEMENTARY CLINICAL FILE

Reporting Summary

## Data Availability

The authors declare that all data supporting the findings of this study are available within the paper and its supplementary information (supplementary data and supplementary clinical files) itself.
