# Supplementary material for: Polyfunctional CD4 T-cells correlating with neutralising antibody is a hallmark of COVISHIELDTM and COVAXIN® induced immunity in COVID-19 exposed Indians
Source: NPJ Vaccines. 2023 Sep 14;8:134. doi: 10.1038/s41541-023-00731-w (PMC10502007; doi:10.1038/s41541-023-00731-w)
Supplement: Supplementary file 1 — Supplementary Figures and Tables [file 41541_2023_731_MOESM1_ESM.pdf]

SUPPLEMENTARY INFORMATION FOR

Polyfunctional CD4+ T-cells correlating with neutralising antibody is a hallmark of COVISHIELD and COVAXIN induced immunity in COVID-19 exposed Indians

Srabanti Rakshit[1], Sudhir Babji[2†], Chaitra Parthiban[1], Ramya Madhavan[2], Vasista Adiga[1,3], Sharon Eveline J[1], Nirutha Chetan[1], Asma Ahmed[1], Sudarshan Shivalingaiah[1], Nandini Shashikumar[1], Mamatha V[4], Avita Rose Johnson[4], Naveen Ramesh[4], Ramkrishna Bhooma Goud[4], Mangaiarkarasi Asokan[2,5], Satyajit Mayor[5], Gagandeep Kang[2], George D'souza[6], Mary Dias[1,4], Annapurna Vyakarnam[1,7]

Supplemental File Contains

Supplementary Figures 1 - 13
Supplementary Tables 1 – 4
Supplementary Methods

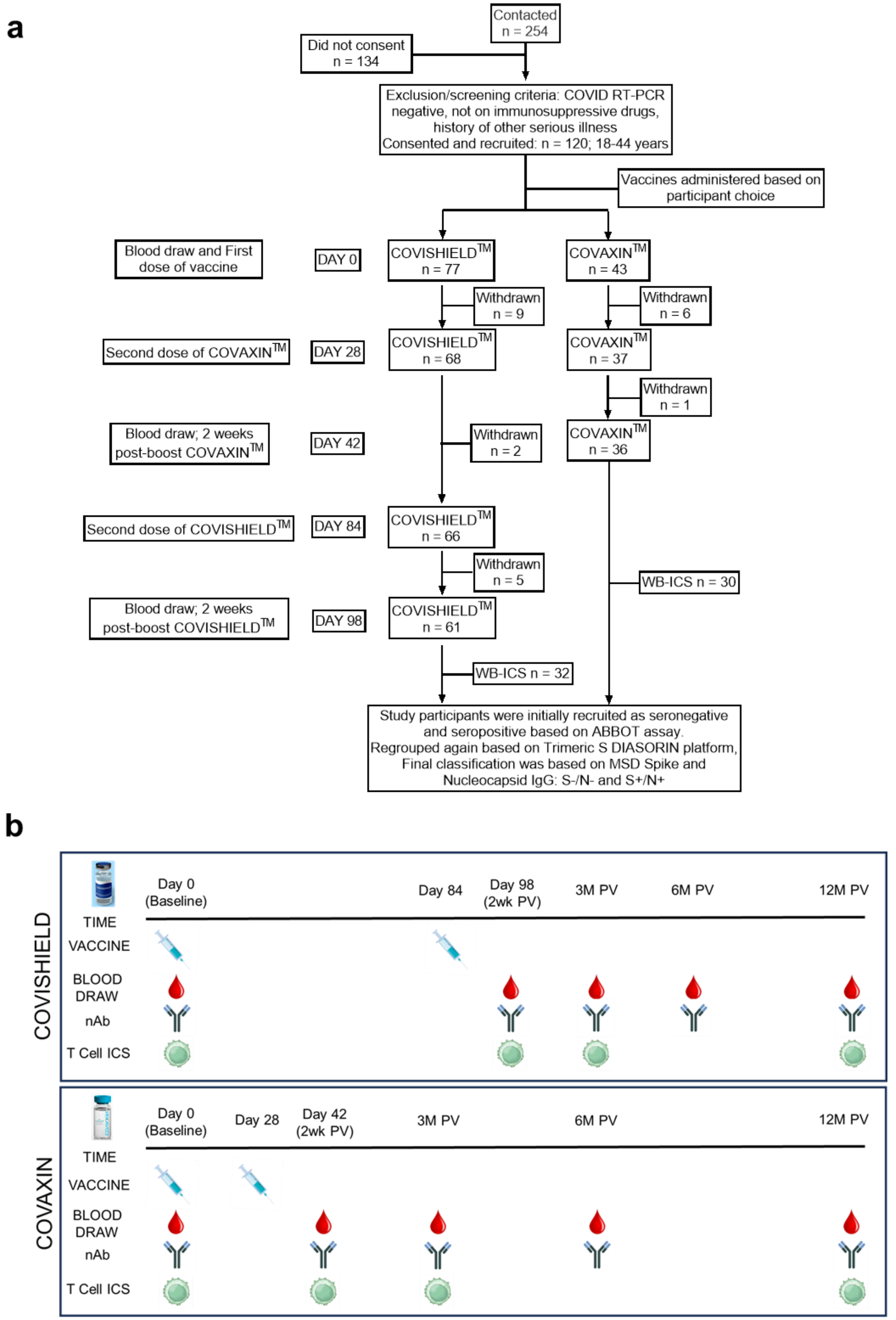

Supplementary Fig. 1: Subject recruitment and study protocol. a Consort flow diagram explaining clinical details of the study: on subject enrolment (n = number of subject) in different vaccine arms. days of blood draw and administration of two vaccine doses of each vaccine. **b** A diagrammatic representation of the study design, including the schedule of COVISHIELD™ and COVAXIN® vaccination, blood draw and analysis of vaccine immunogenicity by antibody and cell-mediated assessments. PV : post-vaccination (after administration of two doses of the primary series).

a

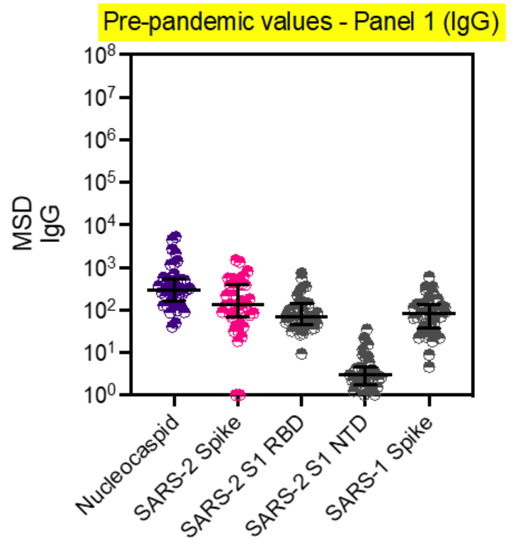

	Nucleocapsid	SARS-2 Spike	SARS-2 S1 RBD	SARS-2 S1 NTD	SARS-1 Spike
Number of values	39	39	39	39	39
Minimum	40.47	1	9.36	1	4.59
25% Percentile	163.7	68.56	44.49	1.71	36.84
Median	298.5	136	69.46	2.96	81.39
75% Percentile	529.4	389.1	142.2	4.51	137.8
Maximum	5242	1488	735.4	35.01	604.4
Mean	704.3	273.6	124.3	5.373	115.3
Std. Deviation	1128	338.4	148.9	6.898	118.6
Mean + 3SD	4088.3	1288.8	571	26.067	471.1

Cut-offs for MSD data

b

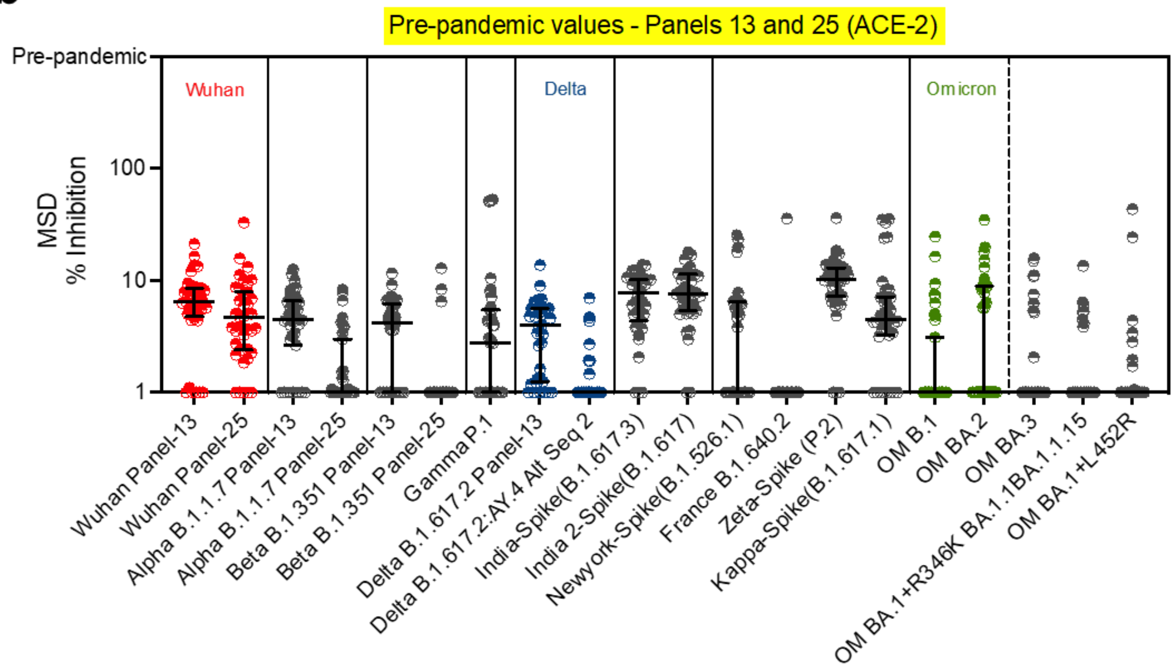

	Wuhan (P-13)	Wuhan (P-25)	Alpha B.1.1.7 (P-13)	Alpha B.1.1.7 (P-25)	Beta B.1.351 (P-13)	Beta B.1.351 (P-25)	Gamma P.1	Delta B.1.617.2	Delta B.1.617.2:AY.4 Alt Seq 2	India B.1.617.3	India 2-B.1.617	Newyork B.1.526.1	France B.1.640.2	Zeta P.2	Kappa B.1.617.1	OM B.1	OM BA.2	OM BA.3	OM BA.1+R346K BA.1.1BA.1.1.15	OM BA.1+L452R
Number of values	39	39	39	39	39	39	39	39	39	39	39	39	39	39	39	39	39	39	39	39
Minimum	1	1	1	1	1	1	1	1	1	1	1	1	1	1	1	1	1	1	1	1
25% Percentile	4.76	2.39	2.65	1	1	1	1	1.25	1	4.37	5.37	1	1	7.18	3.26	1	1	1	1	1
Median	6.44	4.72	4.47	1	4.17	1	2.79	4	1	7.75	7.61	1	1	10.18	4.44	1	1	1	1	1
75% Percentile	9	8	7	3	6	1	5	6	1	10	11	6	1	13	7	3	9	1	1	1
Maximum	21.18	32.94	12.58	8.29	11.58	12.89	52.81	13.74	7	13.88	17.93	25.36	35.71	36.24	35.53	24.67	34.84	15.81	13.52	43.56
Mean	6.904	5.947	4.809	2.071	3.871	1.635	8.03	4.021	1.461	7.417	8.364	5.014	1.89	10.57	7.672	3.025	5.595	2.8	2.049	2.931
Std. Deviation	4.24	5.702	3.024	1.963	2.727	2.352	15.21	2.729	1.237	3.679	4.177	6.257	5.558	5.92	9.281	4.771	7.111	3.961	2.482	7.666
Cut-offs for MSD data																				
Mean + 3SD	20	23	14	8	12	9	54	12	5	18	21	24	19	28	36	17	27	15	9	26

Supplementary Fig. 2: Antibody binding and neutralizing antibody levels measured in pre-pandemic serum samples (n = 39). a Antibody IgG levels to SARS-CoV-2 nucleocapsid, spike, S1 RBD, S1 NTD and SARS-CoV-1 spike protein were measured by MSD V-PLEX COVID-19 Coronavirus Panel 1 (IgG). **b** Neutralizing antibody (% ACE2 inhibition) to ancestral spike, alpha, beta, gamma, delta, zeta, kappa and different omicron variants were measured by MSD V-PLEX SARS-CoV-2 Panel 25 (ACE2). Cut-offs for each assay were determined based on mean + 3 SD values obtained from pre-COVID sera.

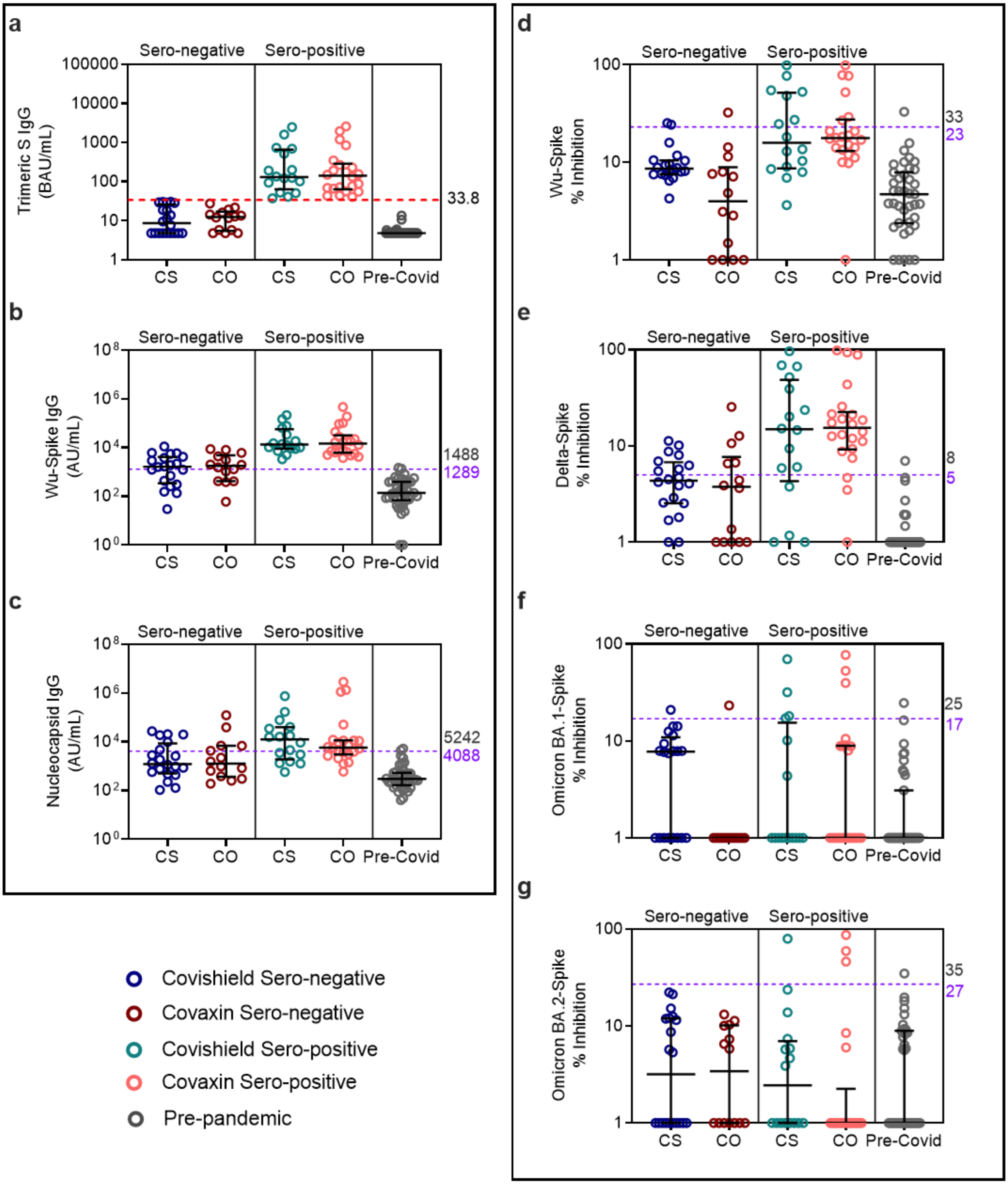

Supplementary Fig. 3: Antibody binding and neutralizing antibody levels against SARS-CoV-2 at baseline in COVISHIELD™ and COVAXIN® vaccinated subjects compared to pre-pandemic samples. **a** IgG against ancestral spike measured on the LIAISON® SARS-CoV-2 TrimericS IgG assay platform with an assay cut-off of 33.8 BAU/ml (horizontal red dotted line). **b-c** SARS-CoV-2 Spike and nucleocapsid IgG titres (AU/ml) measured by MSD V-PLEX COVID-19 Coronavirus Panel 1 (IgG). **d-g** Neutralizing antibody (% inhibition) to Wuhan, Delta and Omicron BA.1 and BA.2 variants measured by MSD V-PLEX SARS-CoV-2 Panel 25 (ACE2). Participants were classified as either seronegative [n=20 for COVISHIELD™ (dark blue); n=14 for COVAXIN® (dark red)] or seropositive [n=16 for COVISHIELD™ (teal), n=22 for COVAXIN® (pink)] at baseline based on TrimericS assay. Dotted lines in purple indicate cut-offs (mean + 3 SD) determined for each SARS-CoV-2 antigen based on pre-COVID sera. Assay cut-offs for each assay are denoted in purple and highest pre-pandemic cut-off in black. Data are shown as median ± IQR.

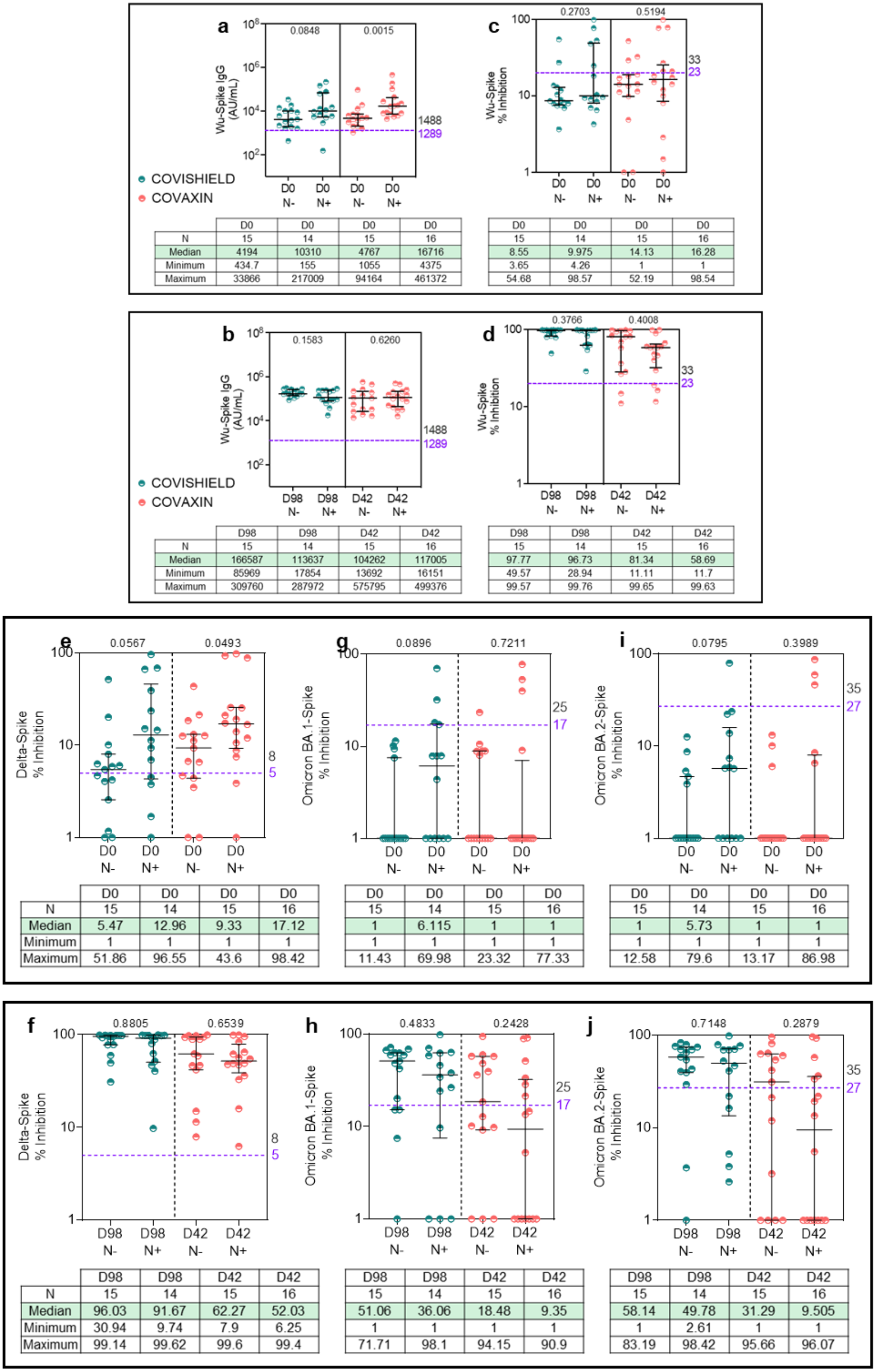

a

	D0	D0	D0	D0
N	15	14	15	16
Median	4194	10310	4767	16716
Minimum	434.7	155	1055	4375
Maximum	33866	217009	94164	461372

c

D0	D0	D0	D0
15	14	15	16
8.55	9.975	14.13	16.28
3.65	4.26	1	1
54.68	98.57	52.19	98.54

b

	D98	D98	D42	D42
N	15	14	15	16
Median	166587	113637	104262	117005
Minimum	85969	17854	13692	16151
Maximum	309760	287972	575795	499376

d

D98	D98	D42	D42
15	14	15	16
97.77	96.73	81.34	58.69
49.57	28.94	11.11	11.7
99.57	99.76	99.65	99.63

e

	D0	D0	D0	D0
N	15	14	15	16
Median	5.47	12.96	9.33	17.12
Minimum	1	1	1	1
Maximum	51.86	96.55	43.6	98.42

g

D0	D0	D0	D0
15	14	15	16
1	6.115	1	1
1	1	1	1
11.43	69.98	23.32	77.33

i

D0	D0	D0	D0
15	14	15	16
1	5.73	1	1
1	1	1	1
12.58	79.6	13.17	86.98

f

	D98	D98	D42	D42
N	15	14	15	16
Median	96.03	91.67	62.27	52.03
Minimum	30.94	9.74	7.9	6.25
Maximum	99.14	99.62	99.6	99.4

h

D98	D98	D42	D42
15	14	15	16
51.06	36.06	18.48	9.35
1	1	1	1
71.71	98.1	94.15	90.9

j

D98	D98	D42	D42
15	14	15	16
58.14	49.78	31.29	9.505
1	2.61	1	1
83.19	98.42	95.66	96.07

Supplementary Fig. 4: Comparison of vaccine-induced antibody responses in COVISHIELD^{TM} and COVAXIN® vaccinees at baseline and 2 weeks post vaccination based on N IgG. a-b IgG levels (AU/ml) and neutralizing antibody (% inhibition) against **c-d** ancestral spike protein, **e-f** delta, **g-h** Omicron BA.1 and **i-j** BA.2 variants were measured in serum samples from COVISHIELD^{TM} and COVAXIN® vaccinees at baseline (D0) and 2 weeks post second dose (D98 for COVISHIELD^{TM} and D42 for COVAXIN®) in the S+/N-/N+ group further segregated as N- and N+. Participants were classified as either N- (n=15) or N+ (n=14) for COVISHIELD^{TM} (teal) and N- (n=15) or N+ (n=16) for COVAXIN® (pink). Scatter plots represent antibody levels and neutralizing antibody (% inhibition). Assay cut-off values for each assay are denoted in purple and highest pre-pandemic cut-off in black. Data is represented as median and IQR. The horizontal bar denotes the median. Statistical analyses was performed using an unpaired Mann-Whitney test.

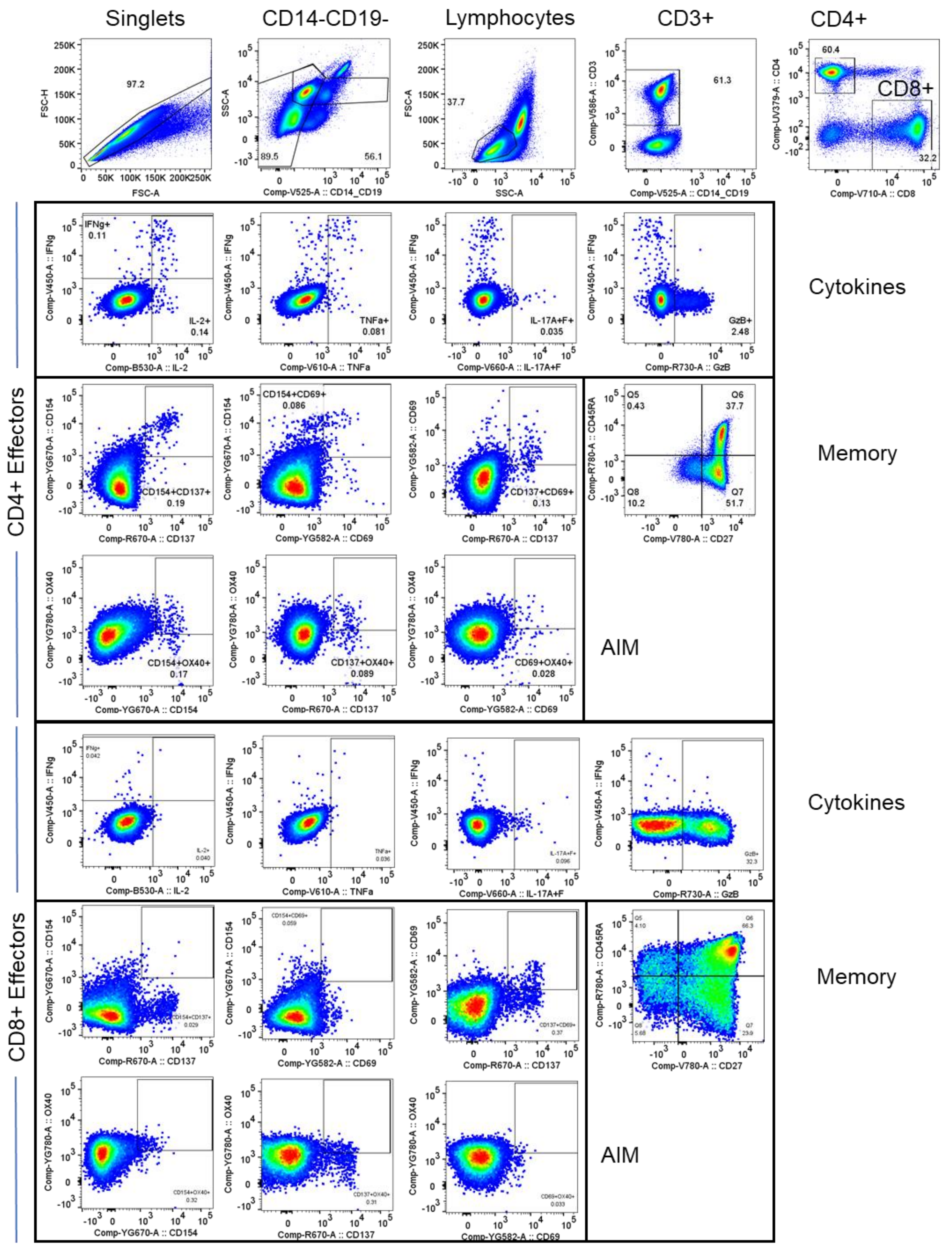

Supplementary Fig. 5: Gating strategy for whole blood T-cell ICS assay that shows representative staining of cytokines, memory and AIM markers included in this study. Spike-specific responses in a COVISHIELD^{TM} vaccinated donor has been shown for both CD4+ and CD8+ T-cells.

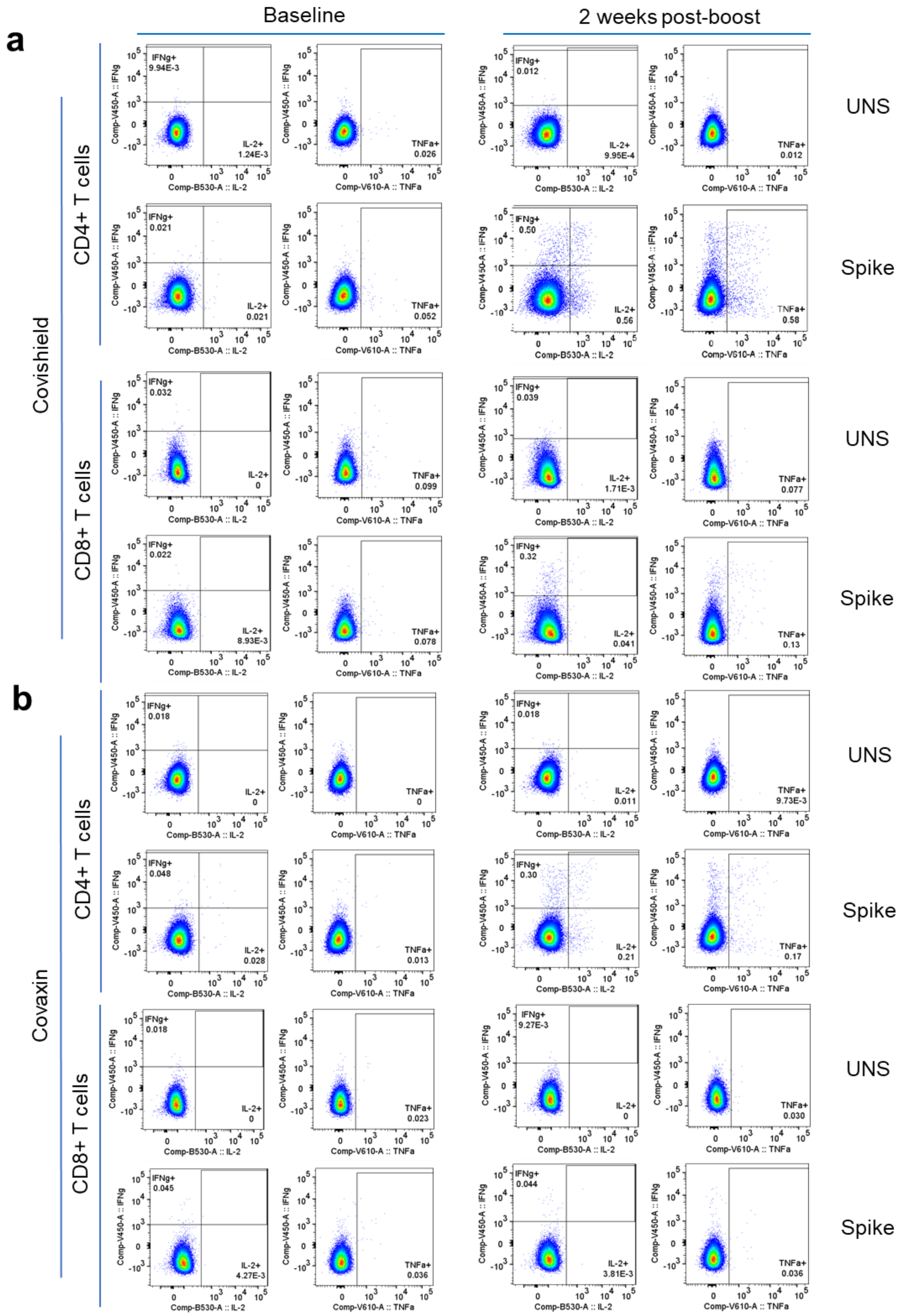

Supplementary Fig. 6: Assessment of SARS-CoV-2-specific T-cells by flow cytometry. Representative FACS plots for the expression of IFN-γ, IL-2 and TNF-α in CD4+ and CD8+ T-cells in whole blood samples from **a** COVISHIELD™ and **b** COVAXIN® vaccinated subjects in response to no peptide (unstimulated) or spike stimulation at baseline and two weeks post-boost are shown.

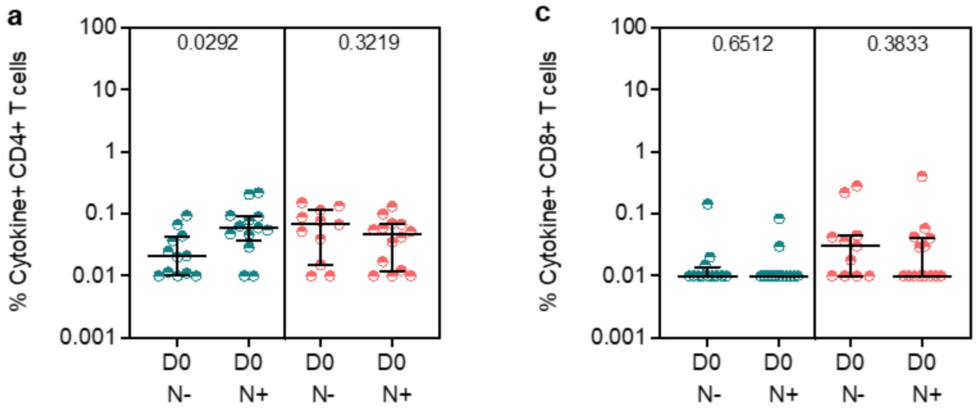

	D0	D0	D0	D0
N	12	13	11	14
Median	0.0205	0.059	0.067	0.047
Minimum	0.01	0.01	0.01	0.01
Maximum	0.094	0.218	0.15	0.1311

D0	D0	D0	D0
12	13	11	14
0.01	0.01	0.03	0.01
0.01	0.01	0.01	0.01
0.144	0.083	0.28	0.4

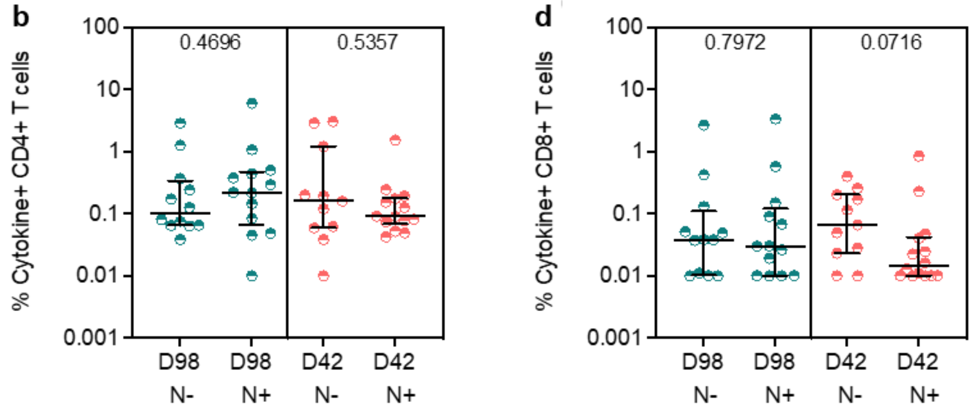

	D98	D98	D42	D42
N	12	13	11	14
Median	0.1025	0.22	0.1585	0.0903
Minimum	0.038	0.01	0.01	0.042
Maximum	2.838	5.956	3.03	1.517

D98	D98	D42	D42
12	13	11	14
0.038	0.03	0.066	0.0145
0.01	0.01	0.01	0.01
2.64	3.326	0.3948	0.844

Supplementary Fig. 7: Comparison of vaccine-induced T-cell responses to ancestral spike at baseline and post vaccination in COVISHIELD™ and COVAXIN® vaccinees based on N IgG. Whole Blood from COVISHIELD™ and COVAXIN® individuals at baseline (D0) and 2 weeks post second dose (D98 for COVISHIELD™ and D42 for COVAXIN®) were stimulated with spike peptide pool (1ug/ml) for 20 hr. **a-b** CD4+ and **c-d** CD8+ T-cells were analyzed for intracellular expression of total cytokine+ cells (IFN-γ or IL-2 or TNF-α) obtained from Boolean gating in FlowJo. Participants were classified as either N- (n=12) or N+ (n=13) for COVISHIELD™ (teal) and N- (n=11) or N+ (n=14) for COVAXIN® (pink). Scatter plots represent background subtracted CD4+ and CD8+ T-cell frequencies. Data is represented as median and IQR. The horizontal bar denotes the median. Statistical analyses was performed using an unpaired Mann-Whitney test.

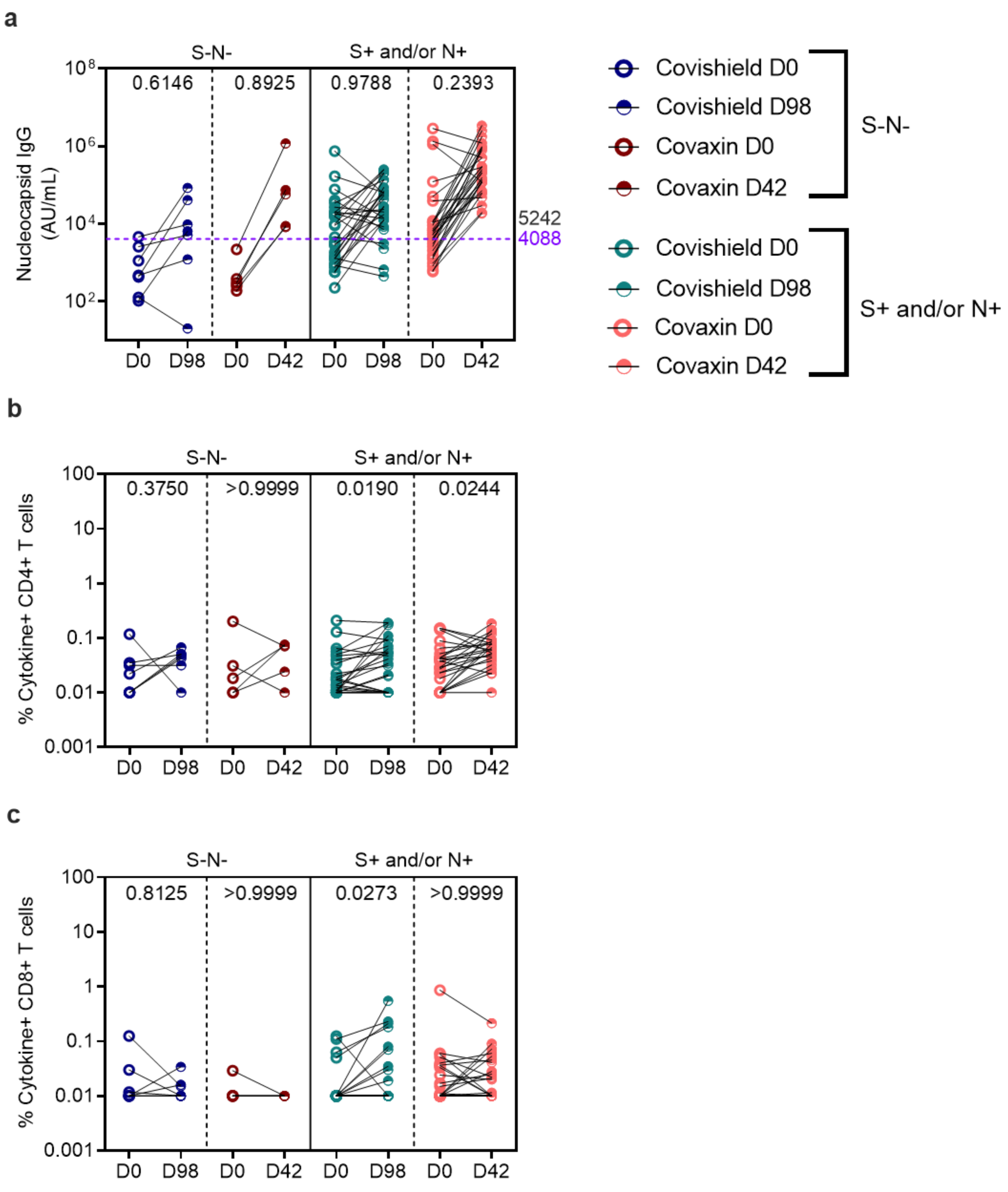

Supplementary Fig. 8: Vaccine-induced antibody and T-cell responses to nucleocapsid. a IgG levels (AU/ml) against nucleocapsid protein in serum samples from COVISHIELD™ and COVAXIN® vaccinees measured by V-PLEX SARS-CoV-2 panel 1 (IgG). Line graphs represent antibody levels at baseline and 2 weeks post-vaccination. Šídák's multiple comparisons test was used for statistical analysis. Whole blood from COVISHIELD™ and COVAXIN® vaccinees at baseline (D0) and 2 weeks post-vaccination (D98 for COVISHIELD™ and D42 for COVAXIN®) were stimulated with nucleocapsid peptide pool (1ug/ml) for 20 hr. **b** CD4+ and **c** CD8+ T-cells were analyzed for intracellular expression of total cytokine+ cells (IFN-γ or IL-2 or TNF-α) obtained from Boolean gating in FlowJo. Line graphs represent background subtracted CD4+ and CD8+ T-cell frequencies expressing effector cytokines. Statistical analyses was performed using a paired Wilcoxon test.

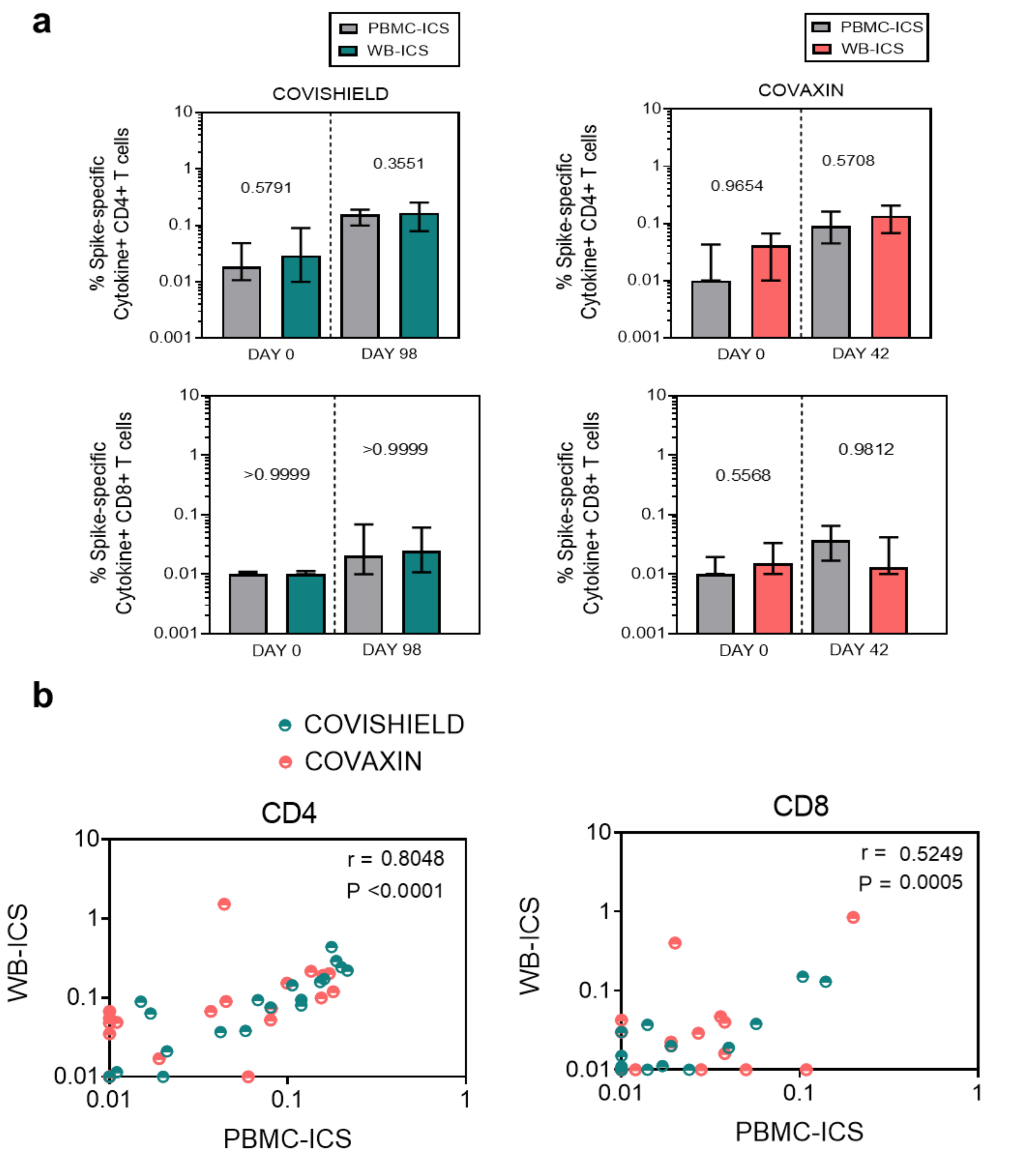

Supplementary Fig. 9: Comparative analysis of whole blood and PBMC-ICS assay reveals similar pattern of vaccine-induced T-cell responses in COVISHIELD^{TM} and COVAXIN® vaccinated subjects. a Frequencies of vaccine-induced total cytokine+ (IFN-γ or IL-2 or TNF-α) CD4+ and CD8+ T-cells at baseline and two weeks post vaccination in response to spike stimulation were compared in whole blood (WB) and matched PBMC samples from N=10 subjects in each vaccinated group. Statistical analyses was performed using One-way ANOVA and Bonferroni's multiple comparisons test. **b** Correlation between WB-ICS and PBMC-ICS assays. Spearman's correlation coefficient (r) and significance values (*P*) are indicated.

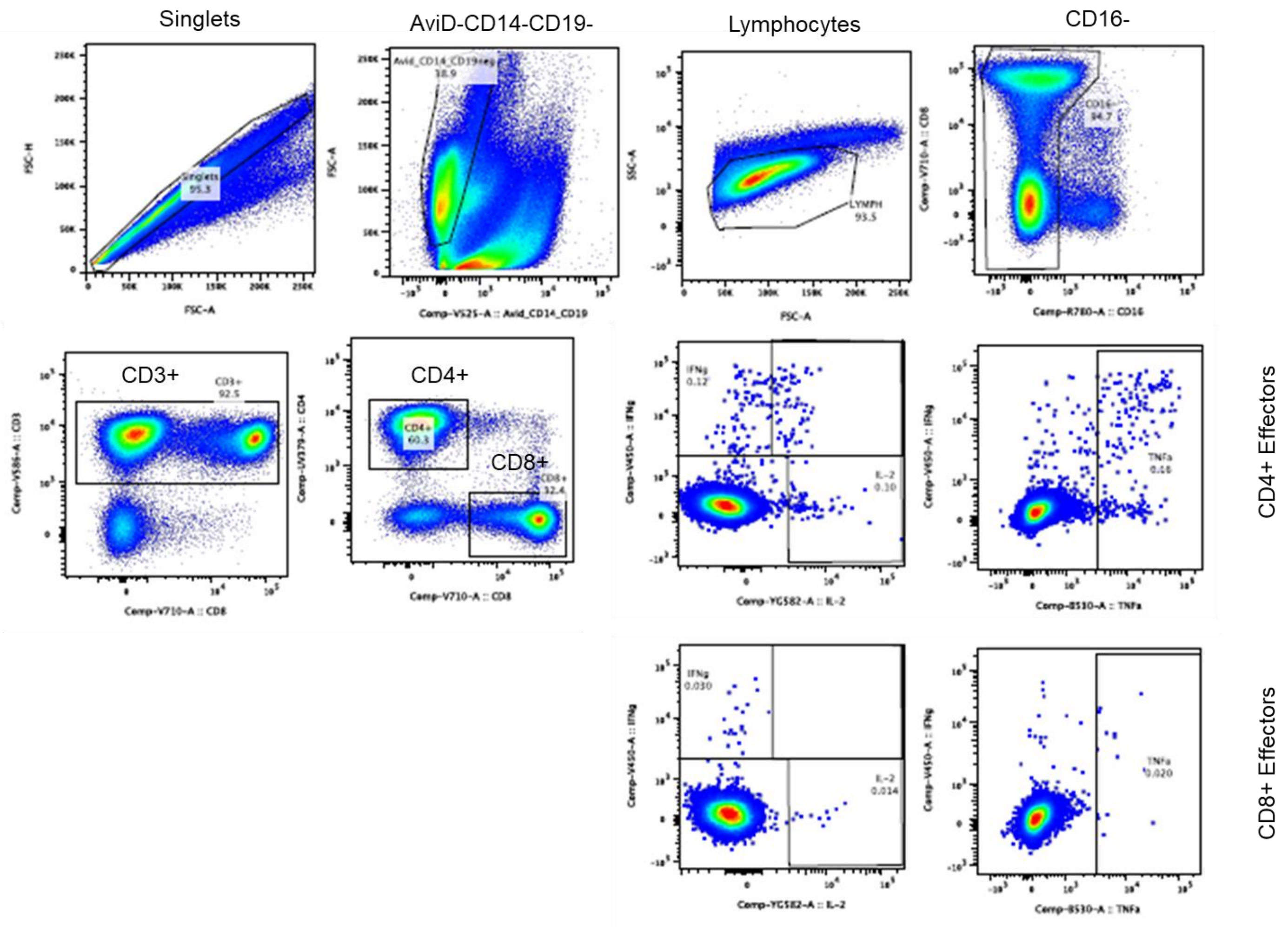

Supplementary Fig. 10: Gating strategy for PBMC T-cell ICS assay that shows representative staining of effector cytokines included in this study. Spike-specific responses in a COVISHIELD^{TM} vaccinated donor has been shown for both CD4+ and CD8+ T-cells.

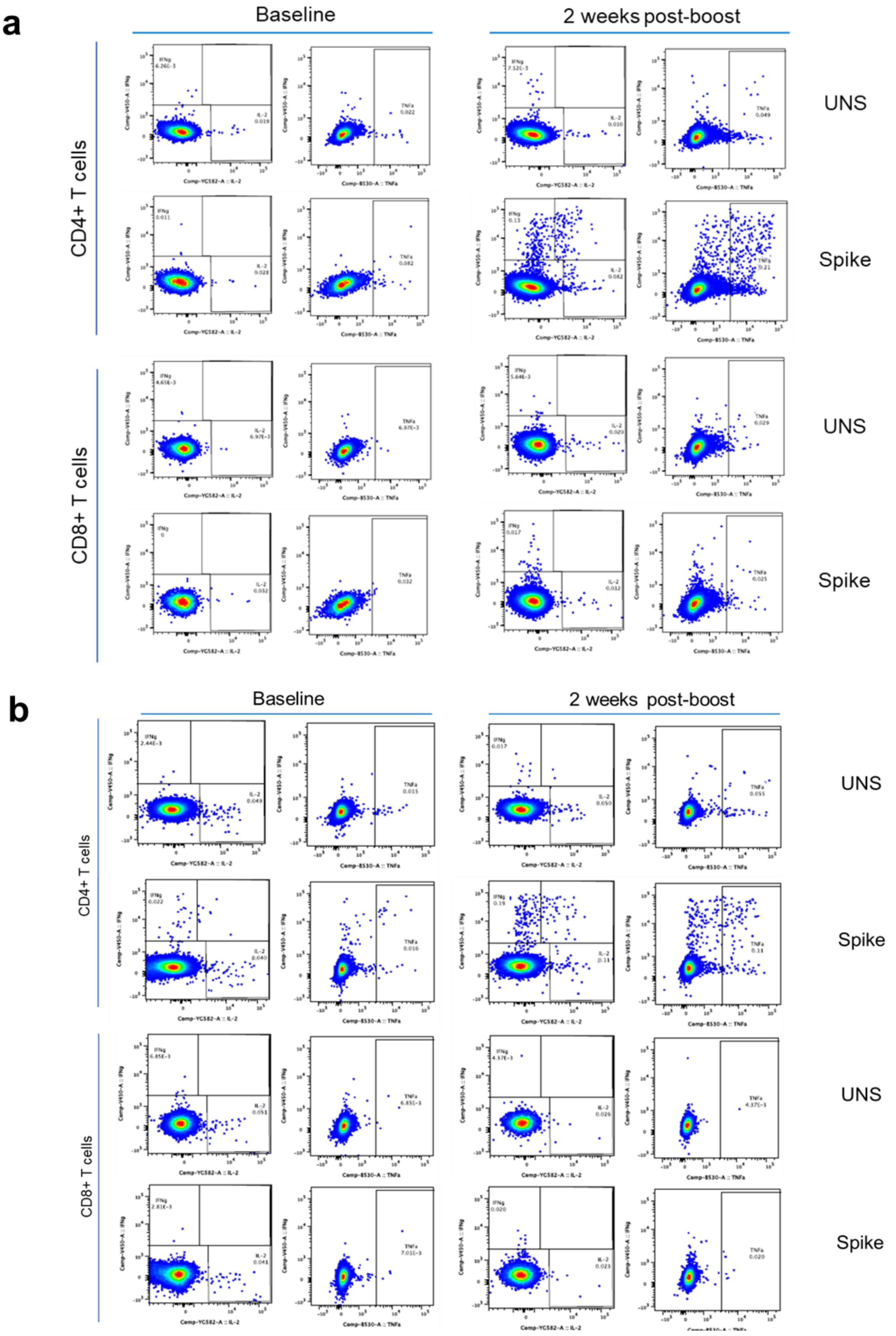

Supplementary Fig. 11: Assessment of SARS-CoV-2-specific T-cells by flow cytometry. Representative FACS plots for the expression of IFN-γ, IL-2 and TNF-α in CD4+ and CD8+ T-cells in PBMC samples from **a** COVISHIELD™ and **b** COVAXIN® vaccinated subjects in response to no peptide (unstimulated) or spike stimulation at baseline and two weeks post-boost are shown.

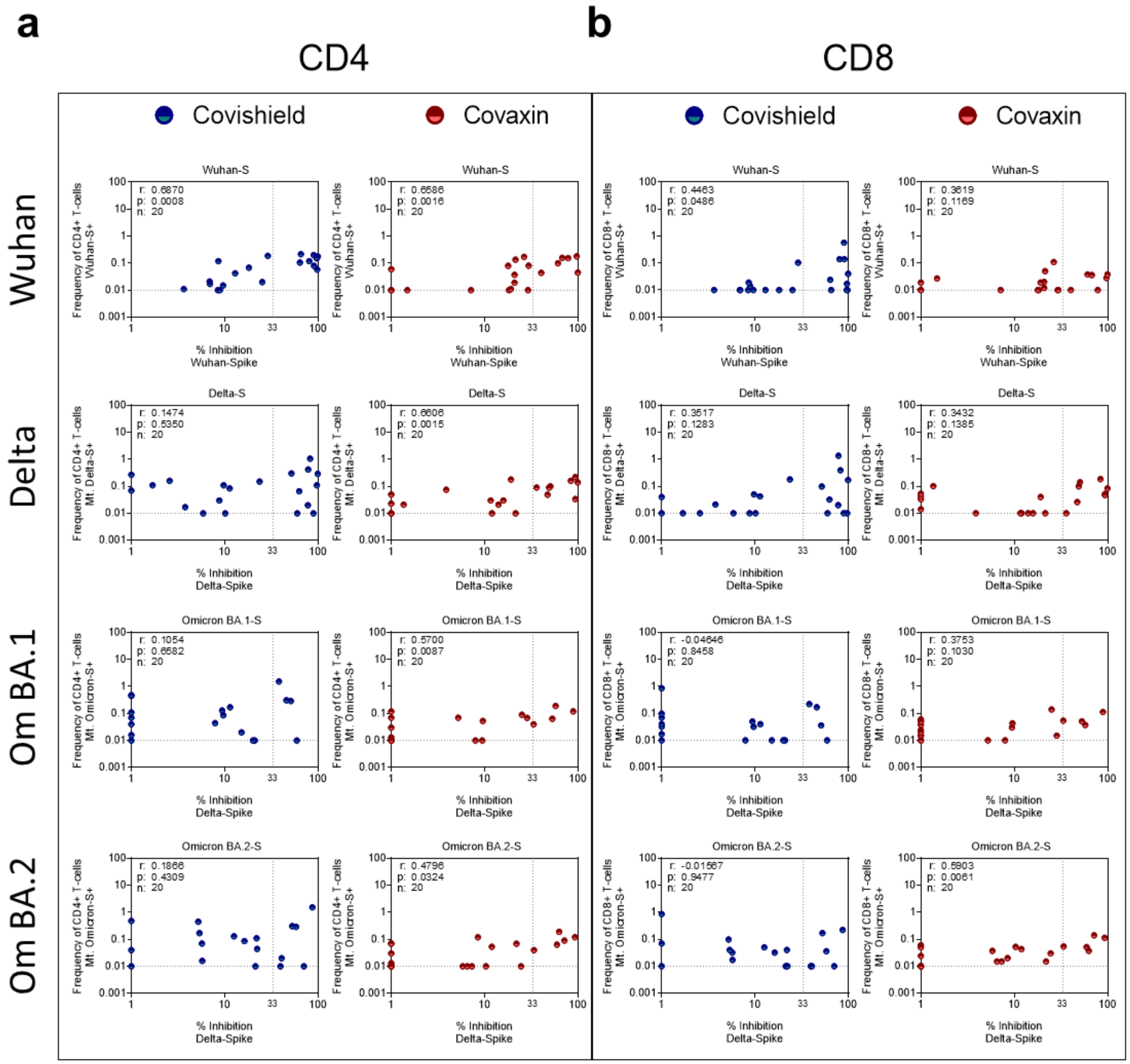

Supplementary Fig. 12: Comparative analysis of vaccine-induced antibody and CD4+ and CD8+ T-cell responses in COVISHIELD™ and COVAXIN® vaccinees. Correlations between frequencies of total cytokine+ **a** CD4+ and **b** CD8+ T-cells (IFN-γ or IL-2 or TNF-α) in response to stimulation of PBMCs with Wuhan, delta and omicron peptide pools and corresponding neutralizing antibody responses (MSD % inhibition) against Wuhan Spike and Delta and Omicron SARS-CoV-2 VOC. Spearman's correlation coefficient (r) and significance values (P) are indicated.

a

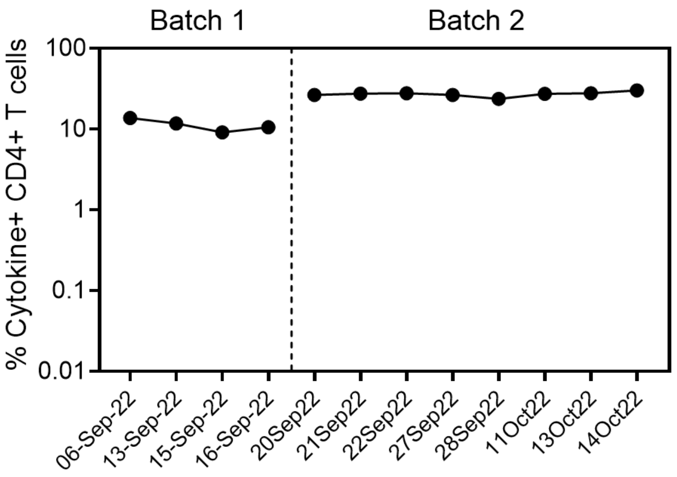

Number of values	4	8
Minimum	9.069	23.55
25% Percentile	9.424	26.33
Median	11.10	27.32
75% Percentile	13.19	27.72
Maximum	13.69	29.98
Coefficient of variation	17.41%	6.675%

b

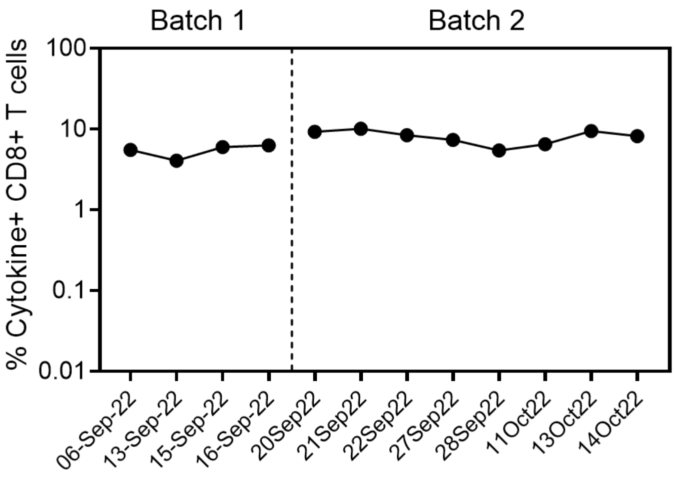

Number of values	4	8
Minimum	4.038	5.410
25% Percentile	4.405	6.685
Median	5.738	8.255
75% Percentile	6.184	9.358
Maximum	6.255	10.05
Coefficient of variation	18.11%	19.53%

Supplementary Fig. 13: Inter-assay variation on measurement of total effector cytokine-expressing CD4+ and CD8+ T-cells by WB-ICS assay. Aliquots of stimulated whole blood samples from a single donor were thawed along with test samples to check for day-to-day variation in ICS staining. Coefficient of variation of PHA-stimulated total cytokine expressing **a** CD4+ and **b** CD8+ T-cells from whole blood were calculated for each day.

SUPPLEMENTARY TABLE 1: Definition of baseline serostatus based on MSD platform

GROUP	ASSAY CUT-OFF
BASELINE SERONEGATIVE (S-/N-)	SPIKE IgG <1289 and<br>Nucleocapsid (N) IgG <4088
BASELINE SEROPOSITIVE<br>(S+ and/or N+)	SPIKE IgG >1289 or<br>N IgG >4088 or<br>SPIKE IgG >1289 and N IgG >4088

SUPPLEMENTARY TABLE 2: Immune parameters pre and post vaccination in subjects designated as seronegative and seropositive at baseline*

ASSAY	ASSAY CUT-OFF	SERONEGATIVE (S-/N-)				SEROPOSITIVE (S+ and/or N+)			
		COVISHIELD™		COVAXIN®		COVISHIELD™		COVAXIN®	
		PRE VAC	POST VAC	PRE VAC	POST VAC	PRE VAC	POST VAC	PRE VAC	POST VAC
Nucleocapsid IgG (MSD)	4088 BAU/ml	472.9	6490↑	303.2	57409↑	4575	28069↑	5446	203472↑
Wuhan Spike IgG (MSD)	1289 BAU/ml	307.9	68791↑	414.6	16882↑	7221	163738↑	7129	104262↑
Neutralizing Ab (MSD) (% inhibition Wuhan Spike)	23%	10.51	86.38↑	3.120	17.86↑	9.400	97.57↑	14.27	58.78↑
Wuhan Spike Cytokine+CD4+ T cell (FCM)	0.01%	0.015	0.15↑	0.023	0.18↑	0.044	0.17↑	0.052	0.12↑
Wuhan Spike Cytokine+CD8+ T cell (FCM)#	0.01%	0.010	0.029↑	0.010	0.010↕	0.010	0.037↑	0.018	0.024↕

* Refer Supplementary Table 1 above.
#FCM: Flow cytometry, ICS assay
↑ Positive response ↕ Neutral response

SUPPLEMENTARY TABLE 3: Antibody panel to track SARS-CoV2 specific T cell responses in whole blood

Sl. No	17C Panel	Cat. No.	Clone	Company	Stain	Dilution
1	CD45RA APC-H7	560674	HI100	BD Pharmingen	CS	1:200
2	CD27 BV785	302832	O323	Biolegend	CS	1:50
3	CD56 BUV737	612767	NCAM16.2	BD Horizon	CS	1:200
4	γδ TCR PE-CF594	562511	B1	BD Horizon	CS	1:200
5	CD14 BV510	301842	M5E2	Biolegend	CS	1:250
6	CD3 BV570	300436	UCHT1	Biolegend	IC	1:50
7	CD4 BUV395	563550	SK3	BD Horizon	IC	1:50
8	CD8 BV711	563677	RPAT8	BD Horizon	IC	1:200
9	IFN-γ V450	560371	B27	BD Horizon	IC	1:100
10	IL-2 FITC	500304	MQ1-17H12	Biolegend	IC	1:50
11	TNF-α BV605	502936	MAb11	Biolegend	IC	1:100
12	IL-17A BV650	563746	N49-653	BD Horizon	IC	1:100
	IL-17F BV650	564264	O33-782	BD Horizon		1:100
13	CD154 PE-Cy5	310808	24-31	Biolegend	IC	1:50
14	CD137 APC	550890	4B4-1	BD Pharmingen	IC	1:12.5
15	CD69 PE	555531	FN50	BD Pharmingen	IC	1:40
16	Ox40 PE-Cy7	350012	Ber-ACT35	Biolegend	IC	1:400
17	GzB Alx 700	560213	GB11	BD Pharmingen	IC	1:100

SUPPLEMENTARY TABLE 4: Antibody panel to track SARS-CoV2 specific T cell responses in PBMC

S. No	9C Panel	Cat. No.	Clone	Company	Stain	Dilution
1	AviD Live/Dead	L34957	NA	ThermoFisher	CS	1:400
2	CD14 BV510	301842	M5E2	Biolegend	CS	1:250
	CD19 BV510	302242	HIB19	Biolegend	CS	1:250
3	CD16 APC-H7	560195	3G8	BD Pharmingen	CS	1:200
4	CD3 BV570	300436	UCHT1	Biolegend	IC	1:50
5	CD4 BUV395	563550	SK3	BD Horizon	IC	1:50
6	CD8 BV711	563677	RPAT8	BD Horizon	IC	1:200
7	IFN-γ V450	560371	B27	BD Horizon	IC	1:100
8	IL-2 PE	559334	MQ1-17H12	BD Pharmingen	IC	1:20
9	TNF-α FITC	11-7349-82	MAb11	eBioscience	IC	1:100
