# Supplementary material for: Polyfunctional CD4 T-cells correlating with neutralising antibody is a hallmark of COVISHIELDTM and COVAXIN® induced immunity in COVID-19 exposed Indians
Source: NPJ Vaccines. 2023 Sep 14;8:134. doi: 10.1038/s41541-023-00731-w (PMC10502007; doi:10.1038/s41541-023-00731-w)
Supplement: Supplementary file 3 — Reporting Summary [file 41541_2023_731_MOESM3_ESM.pdf]

nature portfolio

Corresponding author(s): Annapurna Vyakarnam

Last updated by author(s): 2023/08/12

Reporting Summary

Nature Portfolio wishes to improve the reproducibility of the work that we publish. This form provides structure for consistency and transparency in reporting. For further information on Nature Portfolio policies, see our Editorial Policies and the Editorial Policy Checklist.

Statistics

For all statistical analyses, confirm that the following items are present in the figure legend, table legend, main text, or Methods section.

n/a	Confirmed	
☐	☒	The exact sample size (n) for each experimental group/condition, given as a discrete number and unit of measurement
☐	☒	A statement on whether measurements were taken from distinct samples or whether the same sample was measured repeatedly
☐	☒	The statistical test(s) used AND whether they are one- or two-sided<br>*Only common tests should be described solely by name; describe more complex techniques in the Methods section.*
☒	☐	A description of all covariates tested
☐	☒	A description of any assumptions or corrections, such as tests of normality and adjustment for multiple comparisons
☒	☐	A full description of the statistical parameters including central tendency (e.g. means) or other basic estimates (e.g. regression coefficient) AND variation (e.g. standard deviation) or associated estimates of uncertainty (e.g. confidence intervals)
☒	☐	For null hypothesis testing, the test statistic (e.g. F, t, r) with confidence intervals, effect sizes, degrees of freedom and P value noted<br>*Give P values as exact values whenever suitable.*
☒	☐	For Bayesian analysis, information on the choice of priors and Markov chain Monte Carlo settings
☒	☐	For hierarchical and complex designs, identification of the appropriate level for tests and full reporting of outcomes
☒	☐	Estimates of effect sizes (e.g. Cohen's d, Pearson's r), indicating how they were calculated

Our web collection on statistics for biologists contains articles on many of the points above.

Software and code

Policy information about availability of computer code

Data collection	BD FACSDiva™ version 8.0.1 software was used for acquisition of flow cytometry data.
Data analysis	SPICE 6.1 Pestle 2, FlowJo 10.8.1, OMIQ softwares were used for data analysis.

For manuscripts utilizing custom algorithms or software that are central to the research but not yet described in published literature, software must be made available to editors and reviewers. We strongly encourage code deposition in a community repository (e.g. GitHub). See the Nature Portfolio guidelines for submitting code & software for further information.

Data

Policy information about availability of data

All manuscripts must include a data availability statement. This statement should provide the following information, where applicable:

- Accession codes, unique identifiers, or web links for publicly available datasets
- A description of any restrictions on data availability
- For clinical datasets or third party data, please ensure that the statement adheres to our policy

The authors declare that all data supporting the findings of this study are available within the paper and its supplementary information (supplementary data and supplementary clinical files) itself.

Research involving human participants, their data, or biological material

Policy information about studies with human participants or human data. See also policy information about sex, gender (identity/presentation), and sexual orientation and race, ethnicity and racism.

Reporting on sex and gender	Sex and gender was reported based on self-reporting methods.
Reporting on race, ethnicity, or other socially relevant groupings	The manuscript does not contain identifications by race, ethnicity, or other socially relevant groupings.
Population characteristics	The median age of the study population was 28 years (range, 18–44 years), and 65% were male and 35% were female subjects. The details of the study participants were included in Supplementary File 1.
Recruitment	Ethics approved by the Institutional Ethics Committee (SJRI-298/2021)
Ethics oversight	St. John's Research Institute, Bangalore, India.

Note that full information on the approval of the study protocol must also be provided in the manuscript.

Field-specific reporting

Please select the one below that is the best fit for your research. If you are not sure, read the appropriate sections before making your selection.

☒ Life sciences ☐ Behavioural & social sciences ☐ Ecological, evolutionary & environmental sciences

For a reference copy of the document with all sections, see nature.com/documents/nr-reporting-summary-flat.pdf

Life sciences study design

All studies must disclose on these points even when the disclosure is negative.

Sample size	This study cohort is part of a wider study that involved 8 clinical sites in total and was supported by a CSR funding from Hindustan Unilever Limited (HUL) and Unilever India Pvt. Ltd. (UIPL) and facilitated by the Office of the Principal Scientific Advisor (Government of India). SARS-CoV-2 vaccine-naïve participants were screened for serostatus and recruited at SJRI using a combination of anti-spike and/or anti-nucleocapsid antibodies, either qualitative or quantitative, using kits manufactured by either Roche Diagnostics, Abbott Laboratories or Liaison DiaSorin. Recruitment was done using a combination of unbiased as well serostatus-confirmed inclusion. Baseline samples were re-tested and classified for their serostatus using DiaSorin TrimericS and MSD platforms at Christian Medical College (CMC), Vellore. Sample size calculations were not performed and recruitment numbers were based on who consented after screening. Out of n=254 contacted, n=120 consented. All the participants were enrolled after obtaining informed written consent. Participants with a history of medical illness or prior severe COVID-19 that required ventilation or administration of biologics such as convalescent plasma or monoclonal antibodies were excluded.
Data exclusions	No data was excluded from analysis.
Replication	N/A
Randomization	Allocation of participants to vaccine arms was non-randomized and per participant-choice.
Blinding	The investigators were not blinded to the study.

Reporting for specific materials, systems and methods

We require information from authors about some types of materials, experimental systems and methods used in many studies. Here, indicate whether each material, system or method listed is relevant to your study. If you are not sure if a list item applies to your research, read the appropriate section before selecting a response.

Materials & experimental systems

n/a	Involved in the study
☐	☒ Antibodies
☒	☐ Eukaryotic cell lines
☒	☐ Palaeontology and archaeology
☒	☐ Animals and other organisms
☐	☒ Clinical data
☒	☐ Dual use research of concern
☒	☐ Plants

Methods

n/a	Involved in the study
☒	☐ ChIP-seq
☐	☒ Flow cytometry
☒	☐ MRI-based neuroimaging

Antibodies

Antibodies used	Details of all the antibodies used in flow cytometry assay has been mentioned in Supplementary Table 3 and 4.
Validation	Validation of each antibody has been mentioned online in the manufacturer's website.

Clinical data

Policy information about clinical studies
All manuscripts should comply with the ICMJE guidelines for publication of clinical research and a completed CONSORT checklist must be included with all submissions.

Clinical trial registration	Clinical Trial Registry of India (CTRI) (Registration no. CTRI/2021/09/036258)
Study protocol	Study protocol approved by the Institutional Ethics Committee (SJRI-298/2021)
Data collection	All participant data collection conformed with best clinical practices and were done in home visits and primary health care centers. Clinical details of all participants have been provided in Supplementary File 1.
Outcomes	The primary and secondary outcomes were vaccine immunogenicity in a COVID-19 exposed population and persistence of vaccine induced immunity one year after primary series vaccination.

Flow Cytometry

Plots

Confirm that:

☒ The axis labels state the marker and fluorochrome used (e.g. CD4-FITC).

☒ The axis scales are clearly visible. Include numbers along axes only for bottom left plot of group (a 'group' is an analysis of identical markers).

☒ All plots are contour plots with outliers or pseudocolor plots.

☒ A numerical value for number of cells or percentage (with statistics) is provided.

Methodology

Sample preparation	Detailed sample preparation has been included in methods. Whole blood and PBMCs were used for flow cytometry. Serum samples were used for binding and neutralizing antibody measurements.
Instrument	BD FACSAria™ Fusion (BD BIosciences)
Software	BD FACSDiva™ version 8.0.1 software was used for acquisition of flow cytometry data.<br>SPICE 6.1 Pestle 2, FlowJo 10.8.1, OMIQ softwares were used for data analysis.
Cell population abundance	No sorting of samples was performed.
Gating strategy	Gating strategy has been provided in the Supplementary Information (Supplementary Figures 5 and 10)

☒ Tick this box to confirm that a figure exemplifying the gating strategy is provided in the Supplementary Information.
